# Niclosamide suppresses gastric cancer progression through YTHDF2 inhibition-affected lactate metabolic reprogramming

**DOI:** 10.1016/j.isci.2025.113868

**Published:** 2025-10-27

**Authors:** Mosheng Tang, Yiyin Hu, Xiaohui Zhu, Jichen Dai, Rui Li, Xinran Feng, Kangrui Chi, Xinyi Li, Qiaofei Zhang, Ke Chen, Ruoying Cao, Hanqing Zhu, Haoran Huang, Haipeng Wu, Lejia Sun, Tongke Chen, Jun Wu, Huafu Wang, Zhihong Gui

**Affiliations:** 1 Radiotherapy Center, Lishui People’s Hospital, 15 Dazhong Road, Liandu District, Lishui 323000, Zhejiang, China; 2 The Second Affiliated Hospital and Yuying Children’s Hospital of Wenzhou Medical University, Wenzhou 325027, Zhejiang, China; 3 Wenzhou Medical University, Wenzhou 325027, Zhejiang, China; 4 Department of Head and Neck Surgery, Renji Hospital, Shanghai Jiaotong University, Shanghai 200240, China; 5 School of Ophthalmology and Optometry, Eye Hospital, Wenzhou Medical University, Wenzhou 325000, Zhejiang, China; 6 Laboratory Animal Centre, Wenzhou Medical University, Wenzhou 325000, Zhejiang, China; 7 Department of Pathology, Lishui People’s Hospital, Lishui 323000, Zhejiang, China; 8 Department of Pharmac, Lishui People’s Hospital, Lishui 323000, Zhejiang, China; 9 Department of Nephrology, Lishui People’s Hospital, Lishui 323000, Zhejiang, China

**Keywords:** pharmacology, molecular physiology, cancer

## Abstract

Gastric cancer (GC), a highly aggressive tumor with significant mortality, exhibits metabolic reprogramming involving glucose, lipid, and amino acid metabolism. This study employed non-targeted metabolomics and transcriptomics to analyze metabolic alterations in patients with GC, complemented by single-cell RNA-seq to dissect lactate shuttle dynamics and metabolic alterations between tumor epithelial and endothelial cells. The impact of niclosamide and YTHDF2, an m6A methylation regulator, on gastric cancer cell proliferation was investigated both *in vitro* and *in vivo*. Niclosamide disrupted metabolic reprogramming and lactate dynamics within the tumor microenvironment. YTHDF2 emerged as a critical modulator of GC metabolism, with niclosamide suppressing tumors through the YTHDF2-mediated regulation of metabolic genes. Our research revealed comprehensive metabolic alterations in gastric cancer, including upregulated lactate metabolism that promotes tumorigenesis via the lactate shuttle. Niclosamide targets the m6A methylation regulatory protein YTHDF2, which influences genes related to metabolism, indicating its potential as a prospective treatment for GC.

## Highlights

• Multi-omics analysis revealed the lactate shuttle between epithelial and endothelial cells in GC
• YTHDF2 acts as a key m6A regulator linking metabolic gene expression to tumor progression
• Niclosamide inhibits glycolysis metabolism via YTHDF2, showing potential for GC

## Graphical abstract

**Figure:**
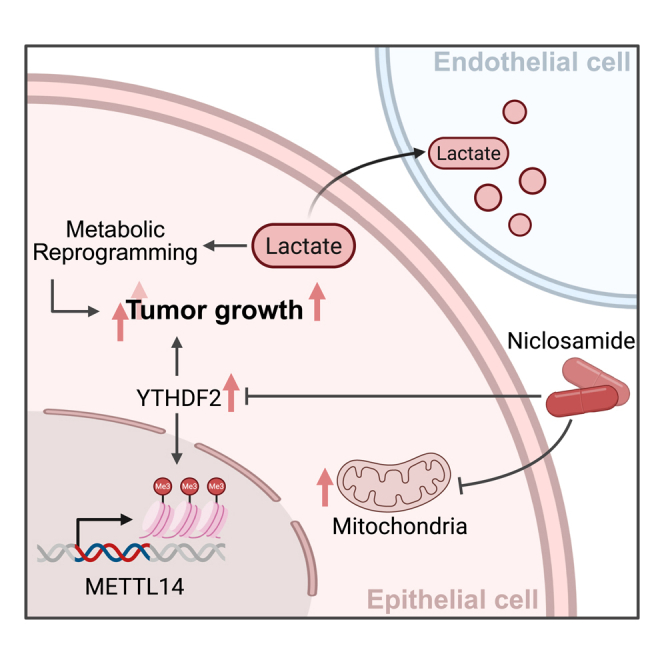

## Introduction

Gastric cancer (GC) is a malignant tumor with a high incidence worldwide, characterized by high mortality rates. Gastric cancer is the third leading cause of cancer-related mortality.^1^ Recent advances in metabolomics and cancer biology have illuminated the critical role of metabolic reprogramming, a process involving dynamic alterations in cellular metabolic pathways, in driving the pathogenesis and progression of gastric cancer. Through adaptive metabolic alterations, gastric cancer cells gain advantages in proliferation, invasion, and resistance to apoptosis, with these metabolic changes being closely associated with their malignancy.^2^ Understanding the intrinsic and extrinsic mechanisms of metabolic changes and the interplay between tumor cell metabolism and the microenvironment’s metabolic dynamics is crucial for developing personalized gastric cancer therapies.

The metabolic alterations observed in gastric cancer are extensive, affecting nearly all major metabolic pathways within the human body, including glucose, lipid, and amino acid metabolism. A hallmark of tumor cell metabolism is aerobic glycolysis, wherein cancer cells absorb substantial amounts of glucose from the tumor microenvironment (TME) to satisfy the elevated energy requirements associated with rapid cell proliferation. This glucose competition effectively suppresses the activation, differentiation, and function of immune cells, thereby facilitating tumor progression.^3^ Furthermore, the dysregulation of lipid metabolism contributes to tumor advancement. Research has shown that gastric cancer tissues demonstrate increased lipogenesis and mitochondrial fatty acid oxidation, as well as elevated fatty acid β-oxidation and lipid peroxidation. This reconfiguration of fatty acid metabolism impacts angiogenesis, thereby promoting tumor cell proliferation.^4^^,^^5^ Additionally, various amino acids, particularly those linked to the citrate cycle (TCA cycle), serve as alternative energy sources for cancer cell proliferation and are significantly more prevalent in gastric cancer tissues.

The Warburg effect is a hallmark of tumor metabolic reprogramming, where tumor cells preferentially undergo glycolysis for energy production despite the availability of oxygen.^6^ Lactate, the main metabolic product of pyruvate through lactate dehydrogenase (LDH), is crucial for tumor cell growth, spread, and immune suppression. Additionally, glutamine metabolism constitutes an alternative source of lactate in tumor cells.^7^^,^^8^ Recent investigations have redefined lactate from being merely a metabolic waste product to a signaling molecule with significant roles in cellular signaling. Research has demonstrated that lactate can induce the liberation of Mg^2+^ from the endoplasmic reticulum, thereby impacting mitochondrial energy metabolism.^9^ Lactate influences fatty acid synthesis by increasing acetyl-CoA carboxylase (ACC) activity.^10^ These findings provide compelling evidence that lactate is intricately involved in cellular metabolism.

Within the tumor microenvironment, a complex network of metabolic pathways interacts to maintain dynamic equilibrium. This network extends beyond the tumor cells themselves, encompassing metabolic symbiosis with stromal cells, including endothelial and immune cells. Research indicates that endothelial cells, in addition to supplying nutrients to tumor tissue, can stimulate tumor stem cells, facilitate metastasis, and modulate the host immune response through paracrine signaling and other mechanisms.^11^^,^^12^ The concept of the lactate shuttle in the TME refers to the transmembrane transport of lactate between different cell populations, facilitating lactate exchange.^13^ Because of the diverse nature of cancer cells and differences in lactate transporter activity (MCT1, MCT4), the lactate shuttle is likely a key facilitator of metabolic symbiosis among cancer cells and between cancer cells and tumor-associated stromal cells.^14^^,^^15^ However, the function of the lactate shuttle between tumor epithelial cells and vascular endothelial cells is not completely comprehended and needs more research.

In the domain of epigenetics, N6-methyladenosine (m6A) modification has been intricately associated with metabolic reprogramming in gastric cancer. Recognized as the most prevalent and ample post-transcriptional modification in eukaryotic mRNA, m6A is integral to the regulation of RNA stability, localization, translation, splicing, and transport.^16^^,^^17^^,^^18^ YTHDF2, a well-characterized m6A reader, demonstrates dual functionalities in both the promotion and inhibition of cancer, thereby presenting significant potential for therapeutic applications in oncology.^19^^,^^20^ It has been documented that YTHDF2 can influence CD8^+^ T cell function by modulating tumor cell glycolysis, consequently affecting tumor progression.^21^ These findings imply that m6A methylation may play a regulatory role in tumor metabolic reprogramming.

Originally used to treat parasitic infections by disrupting oxidative phosphorylation,^22^ niclosamide has emerged as a versatile therapeutic agent for various diseases, such as cancer, viral and bacterial infections, and metabolic disorders. Its mechanism of action involves the modulation of critical signaling pathways such as mTOR,^23^ STAT3,^24^ and NF-κB,^25^ which results in a significant inhibition of tumor cell proliferation and the induction of apoptosis. Furthermore, niclosamide has been shown to modify the metabolic environment and epigenetic landscape of tumors, suggesting its potential as an innovative approach for tumor metabolic therapy.^26^ This study explores the metabolic profile of gastric cancer, examines the lactate shuttle between tumor epithelial and vascular endothelial cells, and investigates the potential of niclosamide, an anti-parasitic drug, in cancer therapy, contributing to improved clinical treatment strategies for gastric cancer.

## Results

### Patients with gastric cancer have global changes in metabolism

**Figure 1:**
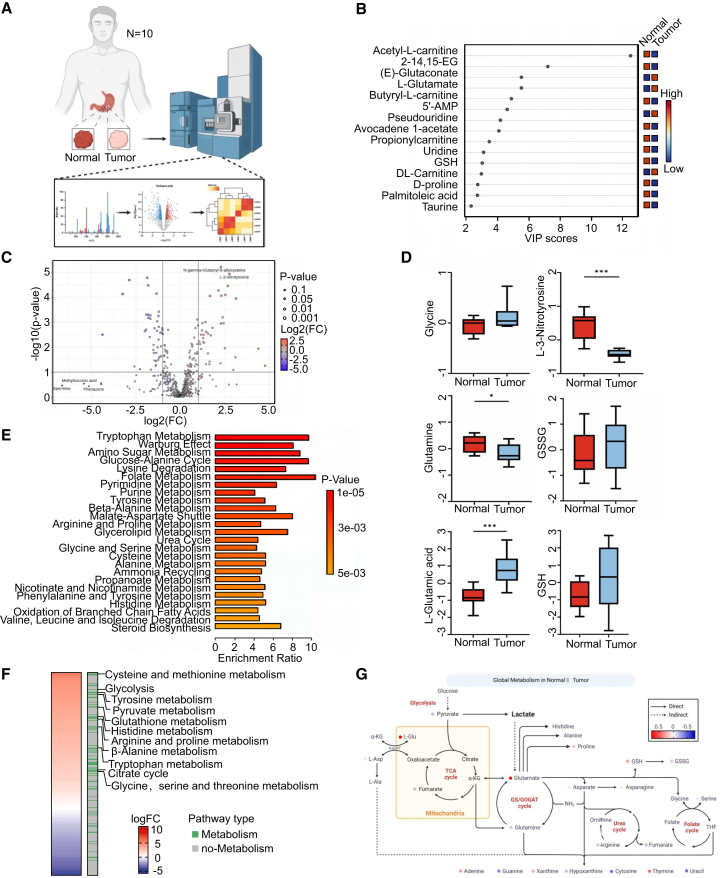
Patients with gastric cancer have global changes in metabolism (A) Schematic diagram of non-targeted metabolomics for detecting metabolic differences in gastric cancer and paracancer tissues (*N* = 10). Samples underwent analysis via LC-MS, with data processed and visualized using multivariate analysis, created with Biorender. (B) The variable importance in projection (VIP) scores identify metabolites that differentiate between normal and tumor samples. (C) Volcano plot depicts differentially abundant metabolites between normal and tumor tissues. Metabolites are highlighted if they have a *p*-value <0.05 and |log2 FC| > 1. (D) Boxplots show the differential abundance of select metabolites (glycine, L-3-nitrotyrosine, GSH, glutamine, L-glutamate, and GSSG) in normal and tumor tissues. Data are represented as mean ± SD. (E) Enrichment analysis of metabolic pathways affected in gastric tumor tissues. (F) Heatmap displays logFC of metabolic pathways in tumor tissue compared to normal tissue. The pathways are ranked by enrichment ratio, and colors represent the type of metabolism (green: metabolism, white: non-metabolism). (G) Schematic representation of the global metabolic network in normal and tumor tissues. Up-regulation and down-regulation are represented in red and blue, respectively, while direct and indirect interactions are depicted with solid and dashed lines, created with Biorender.

To better understand the metabolic traits of gastric adenocarcinoma, we collected 10 paired samples of gastric cancer and adjacent normal tissues from patients. These samples were analyzed using non-targeted metabolomics techniques to identify metabolic differences (Figure 1A). After normalizing the data, partial least squares discriminant analysis (PLS-DA) was utilized for dimensionality reduction, revealing strong biological replicates and notable metabolic differences between the sample groups (Figure S1A). Principal component analysis (PCA) identified the top 15 metabolites with variable importance in projection (VIP) scores over 2, highlighting significant alterations in the levels of metabolites such as glycine, glutamate, glutamine, and glutathione in tumor tissues (Figure 1B). Furthermore, intermediate metabolites or derivatives associated with amino acid metabolism, including N-acetyl-L-glutamine and L-3-nitrotyrosine, were also found to be upregulated in tumor tissues. This highlights the crucial involvement of amino acid metabolism in tumor tissue metabolic processes (Figures 1C and 1D). Pathway enrichment analysis revealed notable changes in amino acid metabolism, particularly glycine, tyrosine, and arginine, alongside alterations in glucose, nucleic acid, folate, nitrogen, and fatty acid metabolism (Figures 1E and S1B). These findings suggest that nearly all major metabolic processes in the human body may undergo remodeling to facilitate tumor progression.

We performed a differential pathway analysis on transcriptomic data from 108 gastric adenocarcinoma samples using dataset GSE26899 (GEO, https://www.ncbi.nlm.nih.gov/geo/query/acc.cgi?acc=GSE26899) to validate the observed transcriptomic changes. The analysis identified significant modifications in several metabolic pathways, notably those involved in amino acid and glucose metabolism, which are consistent with metabolomic findings from human gastric adenocarcinoma tissues (Figure 1F). Importantly, within the pathways enriched from both metabolomic and transcriptomic data, we detected marked upregulation of pathways linked to the Warburg effect, pyruvate metabolism, and galactose metabolism in stomach cancer tissues. This highlights the crucial role of glucose metabolism in providing energy to tumor cells (Figures 1E and 1F).

Based on the above conclusions, we constructed the metabolic landscape of gastric adenocarcinoma and summarized its overall metabolic characteristics. These include augmented glycolysis within tumor cells to meet their elevated energy requirements, an active GS/GOGAT cycle, and nucleic acid metabolism indicative of the high nitrogen demand in tumor tissues for protein and nucleic acid synthesis. Furthermore, the suppression of the urea cycle serves to further enhance glutamine metabolism. Additionally, the upregulation of folate and nucleic acid metabolism is intricately associated with tumor proliferation (Figures 1G and S1C).

### Glucose metabolism and lactate accumulation were reprogrammed in gastric cancer

**Figure 2:**
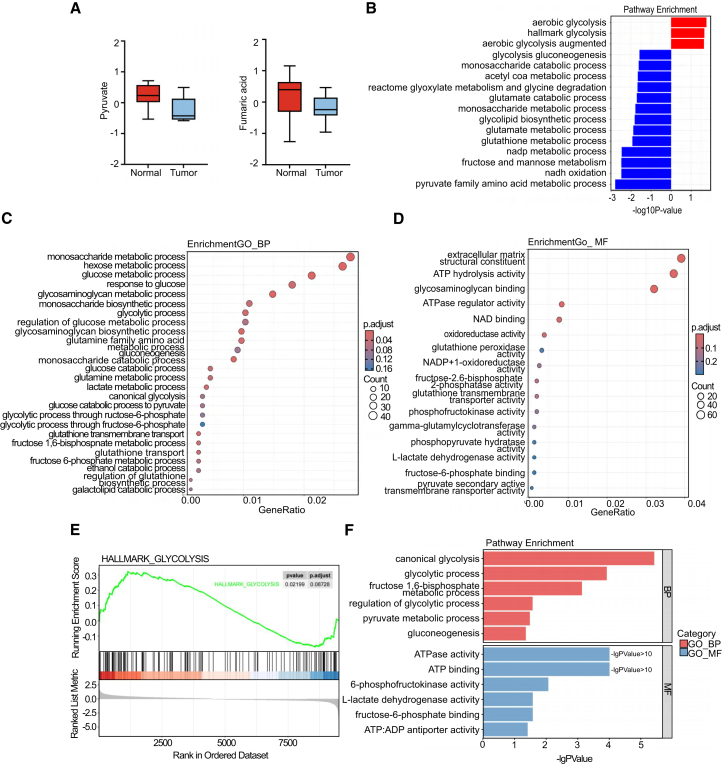
Glucose metabolism and lactic acid metabolism were reprogrammed in gastric cancer (A) Boxplots show the comparison of metabolite levels of pyruvate and fumaric acid between normal and tumor tissues. Data are represented as mean ± SD. (B) Pathway enrichment analysis demonstrated that glycolysis and other key glucose metabolism pathways are up-regulated in gastric adenocarcinoma. (C) Gene ontology (GO) enrichment analysis for biological processes (BP) is visualized with circle sizes indicating the number of enriched genes and colors representing the adjusted *p*-value. (D) GO enrichment analysis for molecular functions (MFs). (E) Glycolysis pathway analysis using GSEA. (F) Summary of pathway enrichment analysis with statistical significance of several glycolytic processes and metabolic pathways.

Based on prior metabolomics analyses, it has been observed that glycolysis, pyruvate metabolism, and other glucose metabolism pathways are markedly enriched in gastric adenocarcinoma tissues. However, the relative concentrations of glycolytic metabolites, such as pyruvate and aconitate, are decreased (Figure 2A). This indicates that, although the glycolytic pathway is upregulated in tumor tissues, certain metabolic processes within the tricarboxylic acid (TCA) cycle may be attenuated. Consequently, tumor cells might divert these metabolites toward alternative pathways to fulfill the material and energy requirements associated with their rapid proliferation, rather than depending solely on the TCA cycle.

We analyzed 134 transcript samples from gastric adenocarcinoma using dataset GSE29272 (GEO, https://www.ncbi.nlm.nih.gov/geo/query/acc.cgi?acc=GSE29272) to further investigate transcriptomic metabolic differences between tumor and normal tissues. The biological differences were corroborated through principal component analysis (PCA) (Figure S1D). KEGG and GO analyses indicate that glucose and its metabolites play a central role in the metabolic reprogramming of gastric cancer (Figures 2B and 2C). Gene set enrichment analysis (GSEA) further supported this finding (Figures 2E and S1E). A notable increase in lactate metabolism was observed, aligning with the Warburg effect, which describes cancer cells' preference for aerobic glycolysis despite oxygen availability (Figure 2C). GO analysis revealed enhanced metabolic activities related to ATP hydrolysis, oxidoreductase activity, and NAD binding in tumor tissues. This suggests that tumors may modulate these energy metabolism pathways to satisfy the heightened energy requirements associated with rapid cellular proliferation. A marked rise in lactate dehydrogenase activity was observed, supporting our metabolomics analysis findings (Figure 2D). Proteomic data from the PRIDE database (PXD033991, https://www.ebi.ac.uk/pride/) corroborated these findings (Figure 2F).

In the oxygen-demanding tumor microenvironment, glucose and galactose are preferentially and rapidly converted to lactate via the glycolytic pathway, rather than being directed into the mitochondria for the tricarboxylic acid (TCA) cycle. Numerous studies have demonstrated that gastric cancer tissues exhibit a significantly elevated lactate microenvironment. Therefore, we hypothesize that lactate may play a critical role in the initiation, progression, and metabolic reprogramming of gastric cancer.

### Development of a metabolism-based prognostic model for gastric cancer and predictive accuracy

**Figure 3:**
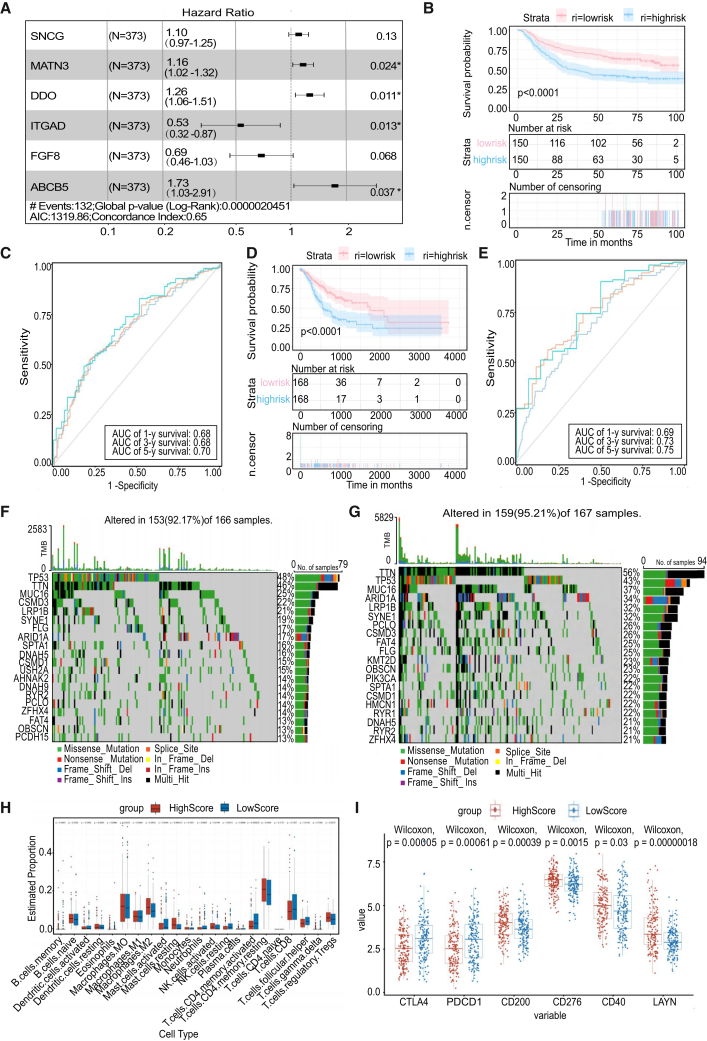
Development of a metabolism-based prognostic model for gastric cancer and predictive accuracy (A) Risk effect estimates among metabolism-related genes by multivariate logistic regression; identification of key genes. (B) Kaplan-Meier survival curves for overall survival (OS) in the training dataset, comparing high-risk and low-risk patient groups. (C) ROC curves and corresponding AUC of the model in training data. (D) Kaplan-Meier survival curves for overall survival (OS) in the validation cohort, comparing high-risk and low-risk groups. (E) ROC curves and corresponding AUC of the model in training data. (F and G) The SNV of high and low risk score groups. (H) Variation in immune cell type abundance between high and low risk score groups. (I) Expression levels of common immune checkpoints, such as PDCD1 and CTLA4, were compared between high and low risk groups.

To further elucidate the role of metabolism in GC and to develop a metabolism-based prognostic model, we initially curated metabolism-related genes from the Genecode database. The TCGA-STAD dataset was used for training, while the GSE26272 dataset served as the validation cohort. In the training cohort, a prognostic model for gastric cancer was developed using univariate and multivariate Cox regression analyses, along with stepwise regression, incorporating the genes SNCG, MNAT3, DDO, ITGAD, FGF8, and ABCB5 (Figure 3A). Based on the median risk score, we categorized the samples into high-risk and low-risk groups. The model demonstrated strong prognostic performance in both the training and validation cohorts (Figures 3B and 3C). The model demonstrated stability and predictive accuracy, with AUC values of 0.68, 0.68, and 0.70 for 1-year, 3-year, and 5-year periods in the training cohort, and 0.69, 0.73, and 0.75 in the validation cohort (Figures 3D and 3E).

Interestingly, SNV analysis showed that the low-risk group exhibited higher mutation levels than the high-risk group, which was unexpected. Previous studies have indicated that high tumor mutational burden (TMB) correlates with improved responses to immunotherapy across various cancers.^27^ Thus, we hypothesize that elevated SNV levels may render the low-risk group as “hot tumors,” whereas diminished SNV levels may characterize the high-risk group as “cold tumors”, potentially resistant to immunotherapy (Figures 3F and 3G). Immune infiltration analyses revealed a significant upregulation of anti-tumor immune cells, including CD4^+^ T cells and CD8^+^ T cells, in the low-risk group. In the high-risk group, there was significant upregulation of resting CD4^+^ T cells and regulatory T cells (Tregs), which are linked to immune suppression. Additionally, the high expression of CTLA4 and PDCD1 in the low-risk group may indicate a heightened sensitivity to PD1-based immunotherapy (Figures 3H and 3I). These findings indicate that the low-risk group exhibits a “hot tumor” phenotype, whereas the high-risk group displays a “cold tumor” phenotype.

### Single-cell profiling reveals risk-associated immune and metabolic alterations in gastric cancer

Intending to explore more about the model genes in the occurrence and development of GC, we downloaded the GC single-cell sequencing dataset GSE163588 from the GEO database, and after quality control and batch effect removal (Figure S2B) from the single-cell sequencing dataset, 54,687 single cells were encompassed in the final dataset, which includes 10 patients with HCC (10 non-tumor liver samples, 10 primary tumor samples, 2 portal vein tumor thrombus (PVTT) samples, and 1 metastatic lymph node sample).

**Figure 4:**
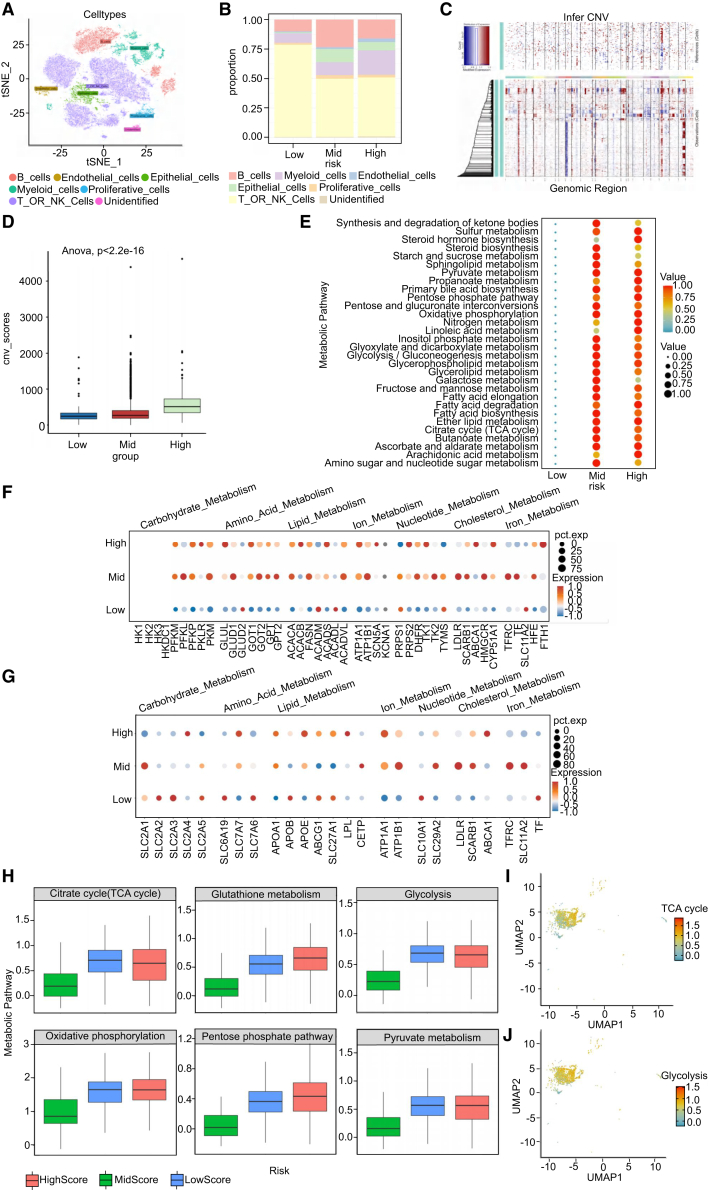
Single-cell profiling reveals risk-associated immune and metabolic alterations in gastric cancer (A) t-Distributed stochastic neighbor embedding (t-SNE) of individual cells, with colors indicating their respective cell lineage or subtype. In t-SNE plots, each point represents an individual cell. (B) The proportion of cells with different cell types in each risk group based on the metabolism-related model. (C) The hierarchical heatmap shows large-scale CNVs in tumor samples. Purple means amplification, and green means deletion. (D) The boxplot illustrates CNV scores for epithelial cells in tumor samples categorized as low, mid, and high risk. InferCNV was used to compute the CNV scores, and ANOVA was employed to calculate the *p* values. (E) scMetabolism package exploration of the metabolic level in each group. (F) The water drop map illustrates the expression levels of enzymes in epithelial cells from samples categorized as high, medium, and low risk. (G) The water drop map illustrates the expression levels of proteins associated with metabolic pathways in epithelial cells from high-, medium-, and low-risk samples. (H) The boxplot illustrates the metabolic levels of glutathione metabolism, glycolysis, and pyruvate metabolism in epithelial cells across high-, medium-, and low-risk samples. (I and J) The UMAP shows the metabolic levels of glycolysis and the TCA cycle of each epithelial cell.

The initial analysis reduced the dimensions of 54,687 cells from the samples, identifying 20 unique tissue states (Figures S2C–S2E). These cell states were then broadly categorized into six major cell types: B cells, epithelial cells, endothelial cells, myeloid cells, proliferative cells, T cells or NK cells, and undefined cells, based on tissue-specific cell markers (Figure 4A). To obtain a deeper insight into the model genes in GC onset and progression, patients were divided into three groups according to the expression levels of SNCG, MATN3, DDO, ITGAD, FGF8, and ABCB5 in their tumor samples: low-risk (LR) (HCC01, HCC05, HCC05, HCC06, HCC07), median risk (MR) (HCC03, HCC08, HCC09, HCC10), high-risk (HR) (HCC02, HCC04)) (Figure S2F). Subsequently, we inspected the distribution of the microenvironment among the three groups. The study observed that as risk increased, the proportion of endothelial and myeloid cells rose, whereas that of T cells and NK cells decreased, confirming alterations in immune cell infiltration within the microenvironment across the three groups (Figure 4B).

To differentiate malignant cells from normal ones, we determined and identified the chromosomal copy number variations (CNVs) of potential epithelial cells in each subgroup using computer-based transcriptome analysis. An inferCNV clustered heatmap was generated for each sample, displaying normalized expression values of immune cells in the top section and ductal cells in the bottom section. In the CNA heatmap, purple represents gain regions, while green denotes loss regions. It came to light that the epithelial cells of tumor samples experienced more significant chromosomal copy number variation (CNV) than normal samples. The notable finding was the increase in CNV score corresponding to higher risk levels (Figures 4C and 4D), indicating a potential link between chromosomal instability and risk degree. Subsequently, we conducted variance analysis among the three subgroups and performed metabolic pathway enrichment analysis on differentially expressed genes (DEGs) using the scMetabolism R package. As shown in the metabolic pathway enrichment analysis, in the mid-score group and high-score group, all metabolic pathways were significantly upregulated, and the enzymes associated with them also showed upregulation (Figures 4E and 4F). Moreover, significant changes were observed in the metabolic pathway-related proteins (Figure 4G). Epithelial cells in high-risk groups exhibit heightened activity in the TCA cycle, glutathione metabolism, glycolysis, the pentose phosphate pathway, oxidative phosphorylation, and pyruvate metabolism (Figures 4H–4J).

### Model gene scores induce altered intercellular communication and metabolic shifts in epithelial and endothelial cells

**Figure 5:**
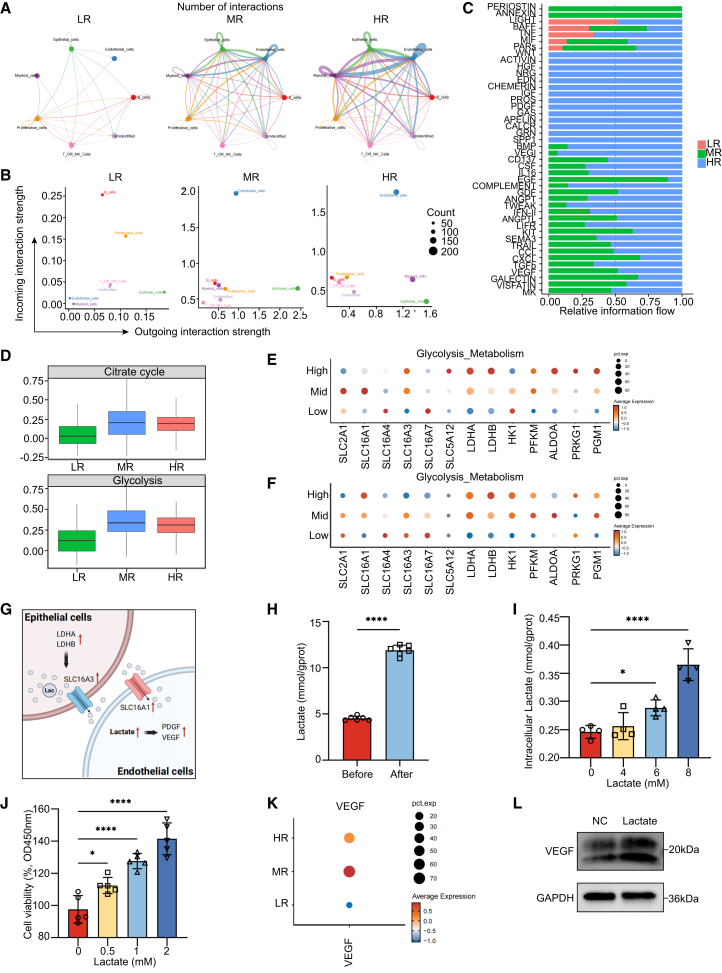
Model gene scores induce altered intercellular communication and metabolic shifts in epithelial and endothelial cells (A) Interaction networks between all cell types in high-, medium-, and low-risk samples. The arrow’s direction denotes the receptor-expressing cell type. Greater interaction weights are represented by a wider edge or larger dot and line. (B) Scatterplots show the interaction strength vs. the number of interactions for each risk group, with cell types color-coded. (C) Bar plot shows the relative information flow across the cell types for each risk group, with color intensity representing the strength of information flow. (D) Boxplots compare expression levels of genes involved in the citrate cycle and glycolysis across LR, MR, and HR groups, with significant differences observed. (E and F) The water drop map shows the expression levels of genes related to glycolysis metabolism in epithelial and endothelial cells. (G) Schematic representation of the lactate shuttle mechanism between epithelial and endothelial cells, created by Biorender. (H) The level of lactate in the culture medium before and after BGC-823 cell culture, Student’s t-Test, ∗∗∗∗*p* < 0.0001. Data are represented as mean ± SD. (I) The intracellular lactate level of HMEC-1 cells treated with lactic acid (4, 6, 8 mM), one-way ANOVA, ∗*p* < 0.05, ∗∗∗∗*p* < 0.0001. Data are represented as mean ± SD. (J) The water drop map shows the expression levels of VEGF of endothelial cells from high-, medium-, and low-risk samples. (K) The CCK-8 assay in HMEC-1 cells, one-way ANOVA, ∗∗∗∗*p* < 0.0001. Data are represented as mean ± SD. (L) Western blot detection of VEGF in HMEC-1.

To further investigate intercellular signaling pathway interactions among cell groups classified by model gene scores, we performed cell communication analysis using the R package CellChat. We observed that as the model score increased, both the number of intercellular interactions and the interaction weight/strength significantly increased (Figures 5A and S3A–S3C). Notably, we found that while the outgoing interaction strength of epithelial cells remained consistently high, the incoming interaction strength of endothelial cells increased significantly with higher model scores (Figure 5B).

Subsequent analysis of signaling pathway changes between epithelial and endothelial cells showed an increased interaction weight/strength between these two cell types (Figure S4A). Further, we identified differentially overexpressed signaling pathways across all cells in the microenvironment. We found that the expression of key components of the VEGF-VEGFR signaling pathway in endothelial cells was significantly elevated in mid- and high-score groups (Figures 5C and S4C), suggesting increased endothelial cell proliferation, as well as enhanced invasion and migration of epithelial cells within tumor tissue. Based on these findings, we extracted endothelial cell subpopulations for further analysis (Figures S3D and S4B) and observed a marked increase in VEGF expression within endothelial cells (Figure S4D).

Examining metabolic changes, we noted significant upregulation of the TCA cycle and glycolysis in endothelial cells within mid- and high-score groups (Figure 5D). Consequently, we analyzed expression changes in glycolytic enzymes and transport proteins in epithelial and endothelial cells across different groups. Both cell types showed the upregulation of LDHA and LDHB expression, suggesting a potential increase in lactate metabolism. In epithelial cells, the elevated expression of SLC16A3 was consistent with lactate export into the microenvironment (Figure 5E), while in endothelial cells, increased SLC16A1 expression might indicate higher lactate exchange with the microenvironment (Figure 5F).

From the observed changes in enzymes and transport proteins in epithelial and endothelial cells, we propose that as the risk score rises, high-risk epithelial cells export lactate into the microenvironment, while endothelial cells increase lactate uptake (Figure 5G). To test this hypothesis, we measured the lactate concentrations in the culture medium before and after culturing BGC-823 cells and found a significant increase, reflecting the phenomenon of high lactate efflux in gastric cancer cells (Figure 5H). Furthermore, we examined the effect of lactate on endothelial cells and observed that lactate exposure increased intracellular lactate levels (Figure 5I) and promoted endothelial cell proliferation (Figure 5J).

This acidic environment may stimulate the upregulation of PDGF and VEGF gene expression in endothelial cells. Additionally, high-risk groups showed the upregulation of ligand-receptor pairs in the VEGF signaling pathway (Figure 5K), which likely contributes to enhanced endothelial cell proliferation, angiogenesis, and metastatic potential in tumor tissue. Meanwhile, Western blot confirmed the upregulation effect of lactate on VEGF (Figure 5L).

### YTHDF2 modulates gastric cancer metabolism and prognosis, correlating with tumor progression

**Figure 6:**
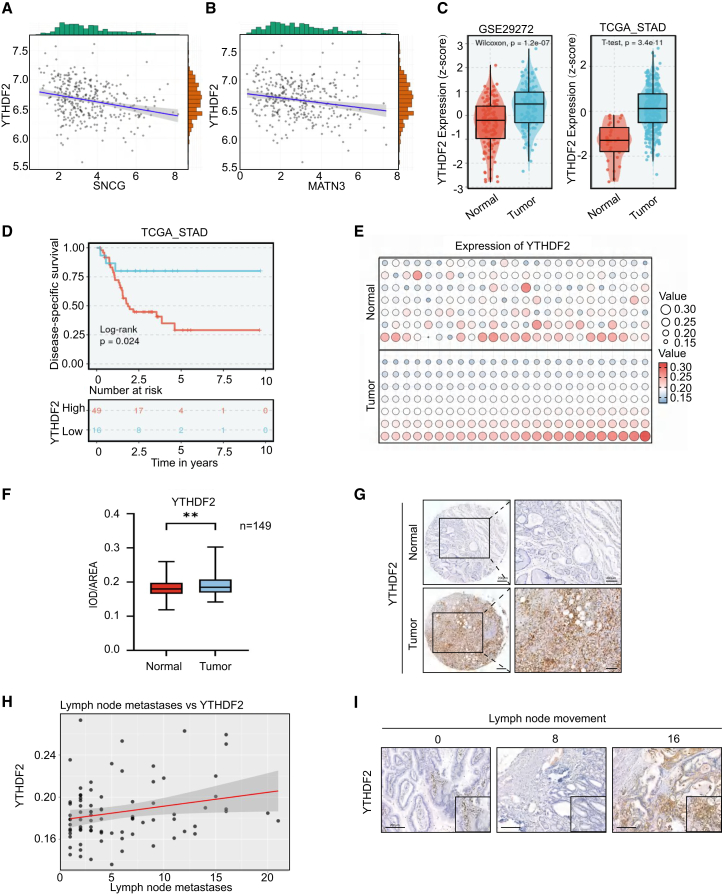
YTHDF2 modulates gastric cancer metabolism and prognosis, correlating with tumor progression (A and B) Correlation analysis of YTHDF2 and SNCG, MATN3. (C) The differential expression of YTHDF2 in normal versus tumor samples was examined using the GSE29272 and TCGA_STAD datasets. (D) Survival analysis of YTHDF2. (E) The bubble chart shows the immunohistochemical positive values of YTHDF2 in normal and tumor tissues. (F) The boxplot shows the relative values of IHC staining for YTHDF2 expression (*n* = 149), Student’s t-Test, ∗∗*p* < 0.01. Data are represented as mean ± SD. (G) Immunohistochemical staining of YTHDF2 in normal and tumor tissues (magnification: 40×). Scale bars, 200 μm and 400 μm. (H) The relationship between YTHDF2 expression levels and the count of lymph node metastases. (I) Immunohistochemical staining of YTHDF2 for different lymph node metastasis conditions (magnification: 40×). Scale bars, 200 μm.

Our research highlights the essential function of YTHDF2 in the metabolism and clinical features of gastric cancer. Correlation analysis revealed that YTHDF2 expression levels are linked to metabolism-related genes such as DDO, ITGAD, FGF8, and ABCB5, with a significant negative correlation with SNCG and MATN3 (r = −0.33 and r = −0.31, *p* < 0.05). The findings suggest that YTHDF2 potentially affects gastric cancer metabolic pathways through gene modulation (Figures 6A, 6B, and S5A–S5D). A comparative analysis of YTHDF2 expression in tumor and normal tissues was conducted using the GSE29272 and TCGA_STAD datasets. The study found that YTHDF2 expression was notably higher in GC tissues than normal tissues (Wilcoxon test *p* = 1.2e-07, T-test *p* = 3.4e-11), indicating its potential effect in the initiation and progression of GC (Figure 6C).

A Kaplan-Meier survival analysis was conducted to investigate the association between elevated YTHDF2 expression and poor prognosis in patients with gastric cancer. Our study revealed that patients with elevated YTHDF2 expression had a conspicuously lower disease-specific survival rate compared to those with lower expression levels (log rank test *p* = 0.024). This indicates a significant link between high levels of YTHDF2 expression and unfavorable outcomes in patients with gastric cancer (Figure 6D). We conducted a tissue microarray analysis on paired samples from 149 patients with gastric cancer to validate the differential expression of YTHDF2 between tumor and normal tissues. The bubble plot (Figure 6E) indicated that about 40% of patients showed notably elevated YTHDF2 expression in tumor tissues. Immunohistochemical staining further supported this observation, showing significantly increased YTHDF2 staining signals in tumor tissues, with the intensity and distribution of staining indicating expression levels (Figures 6F and 6G).

We also checked into the link between increased YTHDF2 expression and gastric cancer metastasis. Scatterplot analysis revealed a strong positive relationship between YTHDF2 expression levels and lymph node metastasis count (r = 0.24), indicating that elevated YTHDF2 expression may facilitate lymphatic metastasis in gastric cancer (Figures 6H and 6I). We also assessed YTHDF2 expression across different stages of gastric cancer (T1-T4) and its relationship with the proliferation marker KI67. The findings revealed that YTHDF2 expression levels increased with disease progression, although this increase was less pronounced at the later stage (T4) compared to earlier stages (Figure S5E). Additionally, the correlation between YTHDF2 and KI67 reinforced its potential involvement in tumor proliferation (Figure S5F). YTHDF2 significantly influences gastric cancer metabolism, metastasis, and prognosis, indicating its potential as a target for therapy.

### Niclosamide inhibits gastric cancer progression by regulating YTHDF2

**Figure 7:**
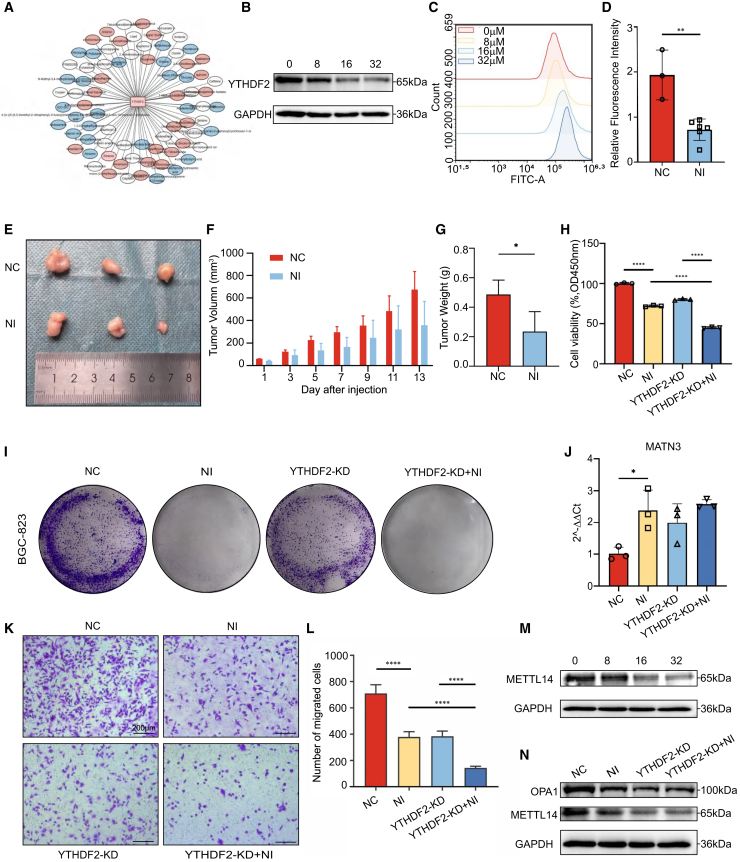
Niclosamide inhibits gastric cancer progression by regulating YTHDF2 (A) Network pharmacology analysis revealed that the drug niclosamide targets YTHDF2. (B) Western blot detection of YTHDF2 in BGC-823. (C) Flow cytometry assesses the effect of varying niclosamide concentrations on cellular ROS levels. (D) The bar chart shows the effect of niclosamide (1 μmol/L) on the proliferation of tumor cells in zebrafish, Student’s t-Test, ∗∗*p* < 0.01. Data are represented as mean ± SD. (E) BGC-823 cells were injected subcutaneously into the right side of the back of BALB/c-cu mice to observe the proliferation ability of the cells. Anatomical tumor images of the control group and the niclosamide treated group (*n* = 3). (F) The tumor volume curve of the control group and the niclosamide treated group. Data are represented as mean ± SD. (G) The tumor weight of the control group and the niclosamide treated group (*n* = 3), Student’s t-Test∗*p* < 0.05. Data are represented as mean ± SD. (H) The CCK8 assay in BGC-823 cells, one-way ANOVA, ∗∗∗∗*p* < 0.0001. Data are represented as mean ± SD. (I) Cell clonal formation experiments were conducted to assess the effect of niclosamide and YTHDF2 knockdown on cell proliferation. (J) Real-time qPCR analysis for MATN3 expression, one-way ANOVA, ∗*p* < 0.05. Data are represented as mean ± SD. (K) The images of transwell migration of cells in each group. Scale bars, 200 μm. (L) Quantitative analysis of cell migration, one-way ANOVA, ∗∗∗∗*p* < 0.0001. Data are represented as mean ± SD. (M) Western blot detection of METTL14 in BGC-823. (N) Western blot detection of OPA1 and METTL14 in BGC-823.

In order to identify efficacious pharmacological agents capable of modulating YTHDF2 and ameliorating gastric cancer progression, we employed network pharmacology analysis. This approach elucidated the multi-target action profile of YTHDF2 when influenced by various compounds. Our findings suggest that niclosamide could be crucial in this regulatory process (Figure 7A). We performed a series of *in vitro* and *in vivo* experiments to validate this hypothesis. We first created a niclosamide concentration gradient to evaluate its effect on cell proliferation in BGC-823 cell lines, determining the IC50 for further study (Figure S5G). As the drug concentration increased, YTHDF2 expression was downregulated in a dose-dependent manner (Figure 7B). The results of the reactive oxygen species (ROS) assay demonstrated that intracellular ROS levels escalated with higher concentrations of niclosamide, suggesting that the anti-cancer efficacy of niclosamide may be linked to oxidative stress (Figure 7C).

Furthermore, to validate the drug’s effects *in vivo*, we administered niclosamide at a dose of 25 mg/kg to BALB/c-nu mice bearing subcutaneous gastric cancer xenografts and found an effective inhibition on tumor proliferation (Figures 7E–7G and S5H). We collected tumor tissues for Western blot detection, and found that niclosamide inhibited the expression of YTHDF2, which indicates that niclosamide suppresses tumor growth as an inhibitor of YTHDF2 (Figure S5I). We also exposed zebrafishs to niclosamide and found the remarkable therapeutic effect (Figures 7D, S5J, and S5K). It is consistent with the results of *in vitro* experiments.

To further explore the regulatory interaction between niclosamide and YTHDF2, we conducted YTHDF2 gene knockdown experiments in conjunction with niclosamide treatment. Cell proliferation and colony formation assays demonstrated that YTHDF2 knockdown and niclosamide treatment each significantly inhibited cell proliferation, with the combination of both treatments showing the greatest inhibitory effect (Figures 7H and 7I). It also regulated metabolism-related genes, including MATN3 (Figures 7J, S5L, and S6A). Furthermore, Transwell assays revealed that both niclosamide treatment and YTHDF2 knockdown (YTHDF2-KD) diminished cell migration and invasion capabilities, suggesting that niclosamide may impede gastric cancer metastasis (Figures 7K and 7L).

Previous studies have proved that YTHDF2, as an m6A “reader,” is crucial in the m6A modification process by recognizing and degrading associated mRNAs. Concurrently, METTL14 serves as a key component in the “writer” process, affecting the recognition and localization of modified sites. Both niclosamide treatment and YTHDF2 knockdown were observed to mediate the downregulation of METTL14, indicating that niclosamide and YTHDF2 may inhibit cell proliferation through the regulation of m6A modification (Figures 7M, 7N, and S6E–S6H). Furthermore, our findings revealed a concurrent downregulation of OPA1, a mitochondrial membrane protein, suggesting that niclosamide-induced cell death might be associated with disruptions in mitochondrial fusion and related activities (Figures 7N, S6G, and S6H). Mitochondrial fluorescence imaging corroborated these findings by demonstrating a decrease in mitochondrial number and evidence of mitochondrial dysfunction (Figure S6B). Flow cytometry analysis further substantiated these observations, showing an increase in cell apoptosis in response to escalating niclosamide concentrations and YTHDF2 knockdown, thereby impeding the progression of gastric cancer (Figures S6C and S6D).

### Niclosamide inhibits gastric cancer progression by disrupting metabolic reprogramming and lactate dynamics in the TMC

Previous studies have shown that metabolic reprogramming in gastric cancer tissues meets the heightened energy and biosynthetic demands, facilitating tumor progression. Niclosamide has been identified as an effective inhibitor of tumor proliferation in both *in vivo* and *in vitro* studies. Consequently, this study aims to investigate whether niclosamide exerts its therapeutic effects by disrupting the metabolic reprogramming of gastric cancer tissues, thereby inhibiting tumor growth.

**Figure 8:**
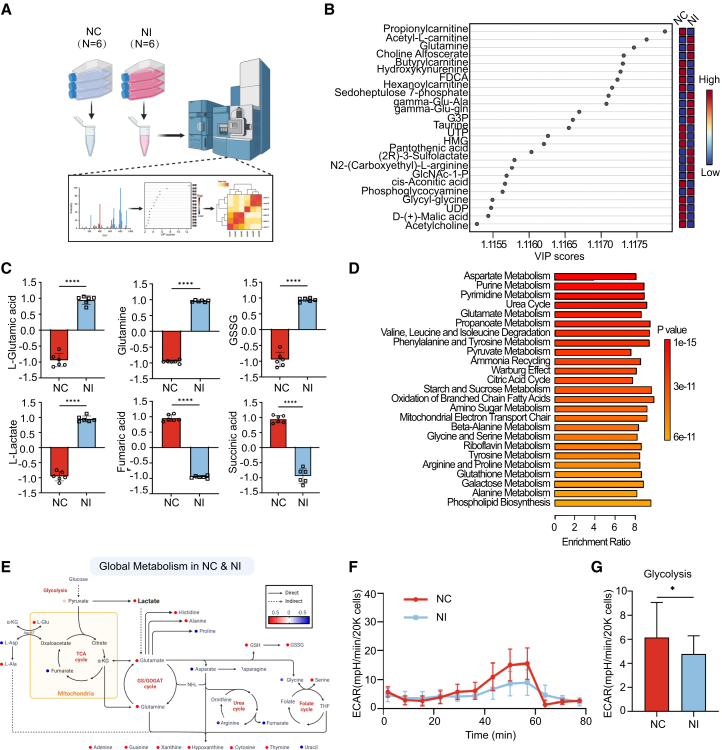
Niclosamide inhibits gastric cancer progression by disrupting metabolic reprogramming and lactate dynamics in the TME (A) Schematic of non-targeted metabolomics illustrates the detection of metabolic differences between the control and niclosamide treatment groups (*N* = 6), created with Biorender. (B) VIP scores of metabolites distinguishing NC and Ni samples. (C) Boxplots show the differential abundance of select metabolites (L-glutamic acid, glutamine, GSSG, L-lactate, fumaric acid, succinic acid) in normal and tumor tissues, Student’s t-Test, ∗∗∗∗*p* < 0.0001. Data are represented as mean ± SD. (D) Enrichment analysis of metabolic pathways between NC and Ni. (E) Schematic representation of the global metabolic network in NC and Ni, created with Biorender. (F) ECAR of control and niclosamide-treated BGC-823 cells was measured at 2×10^4^ cells/well. The Seahorse automatically filled each well with 10 mmol/L of glucose, 1 μmol/L of oligomycin, and 50 mmol/L of 2-DG successively. (G) The glycolysis level of the control group and the niclosamic treated group, Student’s t-Test, ∗*p* < 0.05.

To validate this hypothesis, we administered niclosamide to gastric cancer cells and conducted non-targeted metabolomics to elucidate the metabolic landscape of these cells under the influence of the drug (Figure 8A). Following data normalization and principal component analysis (PCA), the pathway enrichment analysis revealed significant enrichment of metabolic pathways associated with energy supply, including glucose metabolism, glutamine metabolism, and glutathione metabolism, subsequent to niclosamide treatment. Alterations in the Warburg effect and the citric acid cycle suggest that lactate metabolism may be crucial in this process (Figure S6I). Specifically, niclosamide appears to modulate lactate shuttling by altering lactate concentrations in the microenvironment, thereby impacting the progression of gastric cancer (Figure 8C). We analyzed the changes in relevant metabolites, with the volcano plot indicating that metabolites associated with energy metabolism, including glutamine and aspartate, exhibited either upregulation or downregulation. These metabolites were subsequently ranked according to their VIP scores (Figures 8B and S6J). Furthermore, our analysis of additional key metabolites revealed significant changes, strongly suggesting that niclosamide treatment modulates processes such as glucose and energy metabolism. Notably, the observed increase in intracellular lactate levels implies that niclosamide may disrupt the dynamic equilibrium of lactate within and outside tumor cells, thereby inhibiting lactate efflux and reducing lactate concentrations in the microenvironment. This finding corroborates our previous results, which indicated that lactate shuttling between epithelial and endothelial cells may affect the lactate concentration in the tumor microenvironment (Figures 8D and 8E). We demonstrated through the Seahorse experiment that niclosamide treatment could reduce the ECAR and glycolysis levels of gastric cancer cells, which was consistent with the aforementioned results (Figures 8F, 8G, and S6L).

In conclusion, lactic acid serves as a critical risk factor influencing the survival and proliferation of tumor cells within the TME. Niclosamide may mitigate tumor progression and malignant transformation, as well as inhibit tumor development, by reducing lactic acid efflux and modulating glucose and energy metabolism.

## Discussion

In this study, we investigated the global metabolic characteristics of patients with gastric cancer and conducted a comprehensive multi-omics analysis by integrating transcriptomics, proteomics, and metabolomics. Notably, we observed significant alterations in lactate metabolism, characterized by the intracellular accumulation of lactate and its shuttling between tumor epithelial cells and endothelial cells. Furthermore, we developed a prognostic model and identified the m6A methylation regulatory protein YTHDF2, which modulates multiple target genes associated with prognosis. *In vitro* and *in vivo* experiments demonstrate that niclosamide, a broad-spectrum anthelmintic, exhibits anti-tumor effects by modulating YTHDF2, expanding its therapeutic applications.

Metabolic reprogramming represents a fundamental mechanism by which tumor cells adapt to their microenvironment and satisfy the heightened metabolic demands necessary for their rapid proliferation.^28^^,^^29^^,^^30^ The Warburg effect, a defining characteristic of tumor metabolic reprogramming, serves as the principal catalyst for lactate accumulation within the tumor microenvironment. Through the application of non-targeted metabolomics on clinical gastric cancer specimens, we identified extensive metabolic alterations and constructed a comprehensive metabolic profile of gastric cancer. Prior research has underscored the elevated activity of energy metabolism pathways, such as glycolysis, fatty acid synthesis, and oxidation, in gastric cancer cells, which are essential for sustaining their energy requirements.^31^^,^^32^^,^^33^ This study focused on lactate, a byproduct of anaerobic glycolysis, to explore the metabolic symbiosis between tumor epithelial and vascular endothelial cells through lactate shuttling, which is crucial for coordinating signal transduction across cells, organs, and tissues. A reduction in MCT4-mediated extracellular lactate efflux disrupts the tumor microenvironment’s metabolic symbiosis, thereby increasing tumor cell sensitivity to cuproptosis. Additionally, endothelial cells facilitate macrophage polarization through an MCT1-dependent mechanism.^34^ Nonetheless, the specific role and function of lactate in the interaction between epithelial and endothelial cells remain unclear. Our findings indicate that with increasing malignancy, epithelial cells export accumulated lactate, whereas endothelial cells enhance their lactate uptake capacity. This acidification of the tumor microenvironment stimulates the upregulation of growth factors such as PDGF and VEGF, which subsequently promote angiogenesis, tumor cell proliferation, and metastasis. The detailed metabolic profiling presented in this study provides novel insights into gastric cancer metabolic biomarkers, offering potential biological targets for early clinical diagnosis.

Furthermore, N6-methyladenosine (m6A) modification is intricately associated with the metabolic reprogramming observed in gastric cancer.^35^^,^^36^ Prior research has demonstrated that m6A modification influences tumor metabolism through the alteration of RNA stability and the modulation of processes such as splicing and translation. Specifically, it regulates the expression levels of critical metabolic enzymes, thereby facilitating the adoption of more efficient metabolic pathways for energy production by tumor cells. For the current study, we identified metabolic genes pertinent to gastric cancer and developed a robust prognostic model. Correlation analysis of these key genes revealed that YTHDF2, an m6A “reader,” is implicated in the regulation of these pathways. Further research confirmed YTHDF2’s crucial role in promoting tumor cell proliferation and invasion in both *in vitro* and *in vivo* settings. Notably, we also observed a potential association between YTHDF2 expression and lymph node metastasis, suggesting that YTHDF2 may contribute to the promotion of tumor metastasis.

Niclosamide, a well-established anti-parasitic agent, has exhibited notable anti-cancer properties across diverse tumor models in recent years.^37^^,^^38^ This study found that niclosamide alters metabolic reprogramming in gastric cancer tissues by suppressing glycolysis and reducing lactate accumulation. This modulation reduces the cancer cells' reliance on glucose, disrupts the metabolic homeostasis of gastric cancer, and inhibits cellular proliferation. Furthermore, we investigated the impact of niclosamide on m6A methylation modifications and associated genes, including the suppression of the high-risk gene YTHDF2 expression. Importantly, METTL14, a protein subject to regulation by YTHDF2 and implicated in methylation modifications, is also directly modulated by niclosamide. Niclosamide may modify intracellular reactive oxygen species (ROS) levels and mitochondrial function, enhancing its potential as a treatment agent for GC. Our findings highlight niclosamide’s significant impact on metabolic reprogramming and growth inhibition in gastric cancer, though additional research is needed to fully understand its metabolic effects and molecular mechanisms. These results highlight its potential as a metabolic regulator, meriting additional investigation in preclinical studies.

Although our research provides valuable insights, it is important to recognize its limitations. The primary issue is that the research on lactate shuttle and metabolic symbiosis has mainly focused on transcriptomic analyses, making validation challenging. Second, the regulatory network of m6A modification is complex and may vary due to tumor heterogeneity. The specific role of niclosamide in modulating gastric cancer metabolism remains to be elucidated. Ongoing studies are needed to analyze its foundational mechanisms and regulatory pathways, which might contribute to the development of more precise targeted treatments for clinical application. In conclusion, our study underscores the critical role of metabolic reprogramming in the progression of gastric cancer and provides a comprehensive analysis of metabolic symbiosis and epigenetic modifications, particularly the interplay between m6A methylation and metabolic regulation. These findings may inform novel strategies for future targeted therapies and drug development in gastric cancer.

## Resource availability

### Lead contact

Further information and requests for resources and reagents should be directed to and will be fulfilled by the lead contact, Zhihong GUI (guigui0716@163.com).

### Materials availability

All unique models generated in this study are available from the lead contact with a completed materials transfer agreement.

### Data and code availability

Uncropped immunoblotting data and unedited microscopy images are uploaded to Mendeley Data (https://data.mendeley.com/datasets/6zbc9grnnf/1). This article does not report original code. Any additional information required to reanalyze the data reported in this article is available from the lead contact upon request.

## Acknowledgments

This work was supported by the Key Research and Development Project of Lishui City (2022ZDYF09), and the Science and Technology Program of Lishui City (2022GYX25). The authors thank the patients, their families, the investigators, and all the staff involved in this study.

## Author contributions

M.T., Y.H., and X.Z. designed and supervised the study; X.F., Y.C., and K.C. performed most of the experiments; K.C., X.L., and P.H. performed the data analysis; J.D., R.L., and H.H. took charge of bioinformatics analyses; Q.H. participated in functional assays; Q.Z. was engaged in animal investigations; T.C. collected the clinical samples; Y.H., X.Z., and J.D. drafted and embellished the article; J.W., H.W., and Z.G. acquired funding to support this work.

## Declaration of interests

The authors declare no competing interests.

## Declaration of generative AI and AI-assisted technologies in the writing process

In the course of preparing this work, the authors employed ChatGPT to refine the language. They reviewed and edited the content, assuming full responsibility for the publication.

## STAR★Methods

### Key resources table

**Table:** 

REAGENT or RESOURCE	SOURCE	IDENTIFIER
**Antibodies**		

Rabbit monoclonal Anti-METTL14	Cell Signaling Technology	Cat# 48699; RRID:AB_3097700
Mouse monoclonal Anti-GAPDH	Proteintech	Cat# 60004-1-Ig; RRID:AB_2107436
Rabbit monoclonal Anti-Beta Actin	Proteintech	Cat# 66009-1-Ig; RRID:AB_2687938
Rabbit monoclonal Anti-YTHDF2	Cell Signaling Technology	Cat# 71283, RRID:AB_3068618
Rabbit monoclonal Anti-OPA1	Proteintech	Cat# 66583-1-Ig, RRID:AB_2881943
VEGFA polyclocal antibody	Proteintech	Cat# CL594-19003, RRID:AB_2919851

**Biological samples**		

Gastric cancer patients' gastric cancer tissues and normal gastric tissues	The First Affiliated Hospital of Wenzhou Medical University	N/A

**Chemicals, peptides, and recombinant proteins**		

Niclosamide	MedChemExpress	Cat# HY-B0497; CAS:50-65-7
2-Deoxy-D-glucose	Beyotime	Cat# ST1024-5g; CAS:154-17-6
L-(+)-Lactic acid	Sangon Biotech	Cat# A504045; Cas:79-33-4

**Critical commercial assays**		

Lactic Acid assay kit	BioLab	SNM184
AceQ qPCR Probe Master Mix	Vazyme	Q112-02
Cell Counting Kit-8	Dojindo	CK04
Annexin V-FITC Apoptosis Detection Kit	Beyotime	C1062L
Reactive Oxygen Species Assay Kit	Beyotime	S0033S

**Deposited data**		

RNA-seq data	Gene Expression Omnibus	GEO: GSE29272
scRNA-seq data	Gene Expression Omnibus	GEO: GSE183904
RNA-seq data	The Cancer Genome Atlas(TCGA)	TCGA: TCGA-STAD
Original data	Mendeley Data	https://data.mendeley.com/datasets/6zbc9grnnf/1

**Experimental models: Cell lines**		

BGC-823	Cell Bank, Chinese Academy of Sciences	N/A
SGC-7901	Cell Bank, Chinese Academy of Sciences	N/A
HMEC-1	Procell System	CL-0576

**Oligonucleotides**		

Primer for SNCG binding site, forward	TSINGKE	ATGGATGTCTTCAAGAAGGG
Primer for SNCG binding site, reverse	TSINGKE	CTCTGTACAACATTCTCCTT
Primer for MATN3 binding site, forward	TSINGKE	TCTCCCGGATAATCGACACTC
Primer for MATN3 binding site, reverse	TSINGKE	CAAGGGTGTGATTCGACCCA
Primer for DDO binding site, forward	TSINGKE	TCCAGATACACCCATTCACACG
Primer for DDO binding site, reverse	TSINGKE	CCAAATGAACACCAGCATCTCC
Primer for ITGAD binding site, forward	TSINGKE	ACGCCACTACAAGGAAATGC
Primer for ITGAD binding site, reverse	TSINGKE	AGGACAAAGGCACATTTGGG
Primer for FDF8 binding site, forward	TSINGKE	TGAGTTGCCTGCTGTTGC
Primer for FDF8 binding site, reverse	TSINGKE	TGAAGGGCGGGTAGTTGAG
Primer for ABCB5 binding site, forward	TSINGKE	TCTGGCCCCTCAAACCTCACC
Primer for ABCB5 binding site, reverse	TSINGKE	TTTCATACCGCCACTGCCAACTC

**Software and algorithms**		

GraphPad Prism 8.0	GraphPad	https://www.graphpad.com/scientific-software/prism/
ImageJ	Open source	https://imagej.nih.gov/ij/download.html
Novo express	Agilent	https://www.agilent.com/
SoftMax Pro	Molecular devics	https://www.moleculardevices.com/
Metaboanalyst 6.0	Open source	https://www.metaboanalyst.ca
Seurat V4	Open source	https://satijalab.org/seurat/
Wave	Agilent	https://www.agilent.com.cn/
Compound Discoverer 3.3	Thermo Fisher	https://thermo.flexnetoperations.com/

### Experimental model and study participants

#### Ethical approval and consent to participate

Human gastric cancer tissues and adjacent normal tissues from gastric cancer patients admitted to the Department of Gastric Surgery, the First Affiliated Hospital of Wenzhou Medical University were collected with the approval of the First Affiliated Hospital of Wenzhou Medical University. Zebrafish were purchased from the National Zebrafish Resource Center. All animal experiments were approved by the Animal Care and Use Committee of Wenzhou Medical University.

#### Human tissue samples

Fresh gastric cancer tumor tissues were gathered from 10 patients with gastric adenocarcinoma for metabolomics analysis. Tissue samples were gathered at the First Affiliated Hospital of Wenzhou Medical University following ethics committee approval. All patients provided written informed consent for the use of their data and tissues.

#### Mice and ethics statement

Five-week-old female BALB/c-nu mice were procured from Beijing Vital River. All mice were housed under specific pathogen-free (SPF) conditions at the Experimental Animal Center of Wenzhou Medical University. The mice were group-housed in a temperature-controlled environment (20°C–25°C) on a 12-h light/dark cycle, with *ad libitum* access to food. They were randomly assigned to a control group and an experimental group for subcutaneous tumor transplantation. Each mouse was inoculated with 1 × 10^∧^7 BGC-823 cells on the right dorsal flank. When tumor volumes reached approximately 100 mm^3^, mice received niclosamide treatment (25 mg/kg). Body weight and tumor volume were measured every other day. The study protocol received approval from the Animal Ethics Committee of Wenzhou Medical University (wydw2025-0161).

#### Zebrafish embryo cultures and injection

Zebrafish from the National Zebrafish Resource Center were used. The animals were reared in a circulating water system at pH7.0–7.5, 28°C, with a photoperiod of 14h light +10h dark. Embryos were collected after natural oviposition and cultured in EM medium.Using zebrafish at 2dpf, 10 nL of transiently transfected GFP plasmid BGC-823 single cell suspension at an injection density of 3×10^7^/mL was placed in the yolk sac of zebrafish. The experimental group was added with niclosamide (1 μmol/L) in the EM medium, and the control group was added with the same volume of drug solvent. The medium was replaced daily, and images were captured using a fluorescence microscope. Quantitative analysis of fluorescence was performed using ImageJ.

#### Cell culture

Human gastric cancer Cell lines BGC-823 and SGC-7901 were obtained from Cell Bank, Chinese Academy of Sciences. Cells were maintained in Dulbecco’s modified Eagle’s medium (GIBCO C11875500BT) with 10% fetal bovine serum (Sigma F7524) and 1 mM L-Glutamine (Beyotime C0212). Cells were maintained in a 37°C incubator with 5% CO2, with media changes every two days.

### Method details

#### Proteomic data analysis

The PXD033991 project data from PRIDE (https://www.ebi.ac.uk/pride/) underwent FDR <0.01 screening to identify statistically significant protein differences. Gene ontology (GO) analysis was performed using DAVID (https://david.ncifcrf.gov/tools.jsp) on the gene list to identify hub genes enriched in biological processes (BPs) and molecular functions (MFs).

#### Bulk RNA-seq

The Cancer Genome Atlas (TCGA) dataset (https://cancergenome.nih.gov/).

We obtained and examined RNA-Seq count data for gastric cancer (STAD) from the TCGA database, comprising 374 cancer samples and 50 adjacent non-cancerous samples, along with their clinical data. Normalized data were utilized for subsequent analyses.

Gene expression database (GEO, https://www.ncbi.nlm.nih.gov/geo/).

We retrieved GSE29272 from the GEO database (https://www.ncbi.nlm.nih.gov/geo/). A total of 134 normal tissue samples and 134 gastric tumor samples were included. The cardia gastric tumor samples were excluded, and 72 normal tissue samples and 72 non-cardia gastric tumor samples were obtained. mRNA profiles were generated on the GPL96 platform. Normalized data were utilized for subsequent analyses.

The R packages GSEA, clusterProfiler, and org.Hs.e.g.,.db facilitated GSEA and Gene Ontology (GO) analysis on significantly different genes, identifying key genes associated with biological processes (BPs), molecular functions (MFs), and cellular components (CCs).

Utilize the BEST tool (https://rookieutopia.hiplot.com.cn/app_direct/BEST/) for survival analysis of genetic correlation datasets in TCGA-STAD, and perform comparative analysis of gene expression using GSE29272 and TCGA-STAD.

#### Single cell RNA-seq

Single cell sequencing data analysis and differentiation of low -, medium -, and high-risk samples.

The single-cell transcriptome dataset GSE183904 was chosen from NCBI’s GEO database and processed with the Seurat software package. Initially, cell quality control was performed on the single-cell data to exclude low-quality cells.Cells exhibiting mitochondrial gene expression over 20% and erythrocyte gene expression exceeding 2% were excluded. The single-cell data was normalized using NormalizeData, followed by the identification of hypervariable genes with FindVariableFeatures. These genes were then normalized with ScaleData, and principal component analysis (PCA) was conducted on them. UMAP dimensionality reduction was performed using the top 20 principal components, resulting in annotations categorized into cell types including epithelial cells, B cells, and T cells. To assign a cell type to each cluster, we scored each cell cluster by the standardized expression of the following classical markers: Epithelial cells (‘PGA3’, ‘LIPF’, ‘TFF1’, ‘MUC5AC’, ‘CDH1’, ‘REG4’, ‘TFF3’, ‘EPCAM’, ‘CDX1’, ‘CDX2’), endothelial cells (‘PGA3’, ‘LIPF’, ‘TFF1’, ‘MUC5AC’, ‘CDH1’, ‘REG4’, 'TFF3', ‘EPCAM’, ‘CDX1’, ‘CDX2’), endothelial cells (‘PLVAP’, ‘ACKR1’), the B cells (‘MS4A1’, ‘CD79A’, ‘CD19’, ‘TNFRSF17’), T cells (‘CD8A’, ‘IL2RA’, ‘CCR7’), NK cells (‘KLRD1’) and myeloid cells (‘S100A8’, ‘FTL’, ‘CSTA’, ‘CD163’, ‘VCAN’, ‘FCN1’, ‘TPSAB1’, ‘TPSB2’, ‘CPA3’, ‘CD1C’, ‘FCER1A’, ‘CLEC10A’), fibroblasts (‘FN1’), and did not identify cells. The pixel type with the highest score was assigned to each cluster. Gastric cancer tissue samples were classified into low, medium, and high risk ground on the correlation between the expression levels of SNCG, MATN3, DDO, FGF8, ABCB5, and prognosis, facilitating further analysis of m6A’s impact on gastric cancer.

#### Copy number variation (CNV)

The R package ‘inferCNV’ was used to calculate somatic large-scale chromosomal CNV scores.Following the data preparation guidelines for scRNA-seq (https://github.com/broadinstitute/inferCNV), the original matrix’s annotations and gene/chromosome location files are required. Normal epithelial cells were chosen as the reference normal cells. The CNV score was calculated as a quadratic sum of CNV areas.

#### Single nucleotide variant (SNV)

The R package VariantAnnotation was used for evaluation and annotation. According to the data (https://github.com/Bioconductor/VariantAnnotation) - prepare scRNA counting - seq primitive matrix and mutation site files and sample comments. We selected normal epithelial cells as the control group. The SNV score was calculated as a comprehensive assessment of the frequency and number of variants within a specific gene or region.

#### Cell chat analysis

To identify potential cell interactions between all cells, we used the R package CellChat to infer predicted cell-to-cell communication. The CellChat algorithm assesses the importance of ligand-receptor interactions between cell types by analyzing the expression of key factors such as stimulatory and inhibitory membrane-bound coreceptors, as well as soluble agonists and antagonists.

#### Scmetabolism analysis

The scRNA-seq data of epithelial cells were extracted from the scRNA-seq ensemble for the next analysis. The R package scMetabolism was employed for KEGG pathway analysis with the parameter metabolism type set to “KEGG”. Simultaneously, essential information was extracted and visualized.

#### SCAP

Retrieve the GSE26899 project from the GEO database (https://www.ncbi.nlm.nih.gov/geo/), which includes RNA-seq data comprising 12 tumor tissue samples and 96 adjacent normal tissue samples. Use R for data preprocessing. The R package KEGGREST was used to obtain KEGG pathway annotations and label metabolic pathways, and the RNA-seq data that had completed the pre-processing were split to obtain the normal and tumor sets, respectively. The sets were analyzed with the R package SCAP, and results were visualized and pathways labeled using the R package ComplexHeatmap.

#### Metabolomics LC-MS analysis

An extraction solution composed of acetonitrile, methanol, and water in a 2:2:1 ratio was prepared at 4°C, using 0.5 μg/mL L-chlorphenylalanine as the internal standard. After centrifugation for 15min, the supernatant was collected and dried to powder, and then resuspended in 10% methanol. Samples were analyzed by ultra-performance liquid chromatography-electrospray mass spectrometry (UHPLC-ESI-MS). Samples and standards were analyzed using an LCMS system with an Acquity HSS T3 column (1.8 μm × 2.1 mm×100 mm, Waters Corporation, Milford, MA, USA) at 35°C. Separation was achieved via liquid chromatography in positive ion mode, employing a mobile phase of H_2_O with 0.1% formic acid and methanol with 0.1% formic acid. The negative ion mode utilized a mobile phase composed of methanol and a mixture of H_2_O with 0.1% formic acid. The mass spectrometer operated in both positive and negative electrospray ionization modes.

The untargeted metabolomics analysis data obtained by LC-MS/MS were imported into Compound 3.3 software for data processing and analysis, and the unknown metabolites were identified by molecular formula prediction, mzCloud database, HMDB and PubChem database. The metabolites were processed using Metaboanalyst 6.0 for standardization and statistical correction, facilitating analyses such as principal component analysis (PCA), enrichment analysis, and pathway analysis.

#### Niclosamide treatment of cell lines

Niclosamide (MCE HY-B0497) was dissolved in DMSO to create a 10 mM stock solution, which was stored at −80°C. Upon reaching 60–70% confluence, cells were exposed to fresh medium containing 8, 16, 32 μmol/L niclosamide for 24 h. Equal volumes of the corresponding solvent were used to treat the control group.

#### Cell transfection

YTHDF2 plasmid DNA was purchased from the Plasmid Bank of Fudan, Shanghai.Lipofectamine 2000 (Invitrogen 11668030) was used for transient transfection of plasmid DNA into BGC-823 cells. 2 μg of plasmid DNA and 3 μL of Lipofectamine 2000 were each diluted in 125 μL of Opti-MEM, combined and incubated at room. The transfection mixture was evenly applied to the cells, which were then collected for further experiments 48 to 72 h post-transfection. The effectiveness and specificity of the knockdown were confirmed by immunoblotting.

#### Western Blotting

Western and IP cell lysates (Beyotime P0013, China) were used to isolate proteins from cells or homogenize tumor tissues to prepare lysates. The homogenate was centrifuged at 15,000 rpm for 15 min at 4°C to collect the supernatant.Denatured proteins (15–30 μg per sample) were separated using 10% SDS-PAGE and transferred to a PVDF membrane (Millipro IPVH00010, USA). Block with 5% skim milk (Solarbio LP0033B, China) for 1.5 to 2h, wash with 1×TBST (Solarbio T1081, China) three times for 10min each, and then with primary antibody diluent (Beyotime P0023A, China). Primary antibodies were diluted and incubated at 4°C for 12 to 18 h. Following incubation, cells underwent three 10-min washes in TBST, were treated with horseradish peroxidase (HRP) (Abclonal AS003, Abclonal AS014) for 1 h using conjugated secondary antibodies, and then washed again. The bands were then detected using an enhanced chemiluminescence detection kit (Biosharp BL520B). GAPDH was used as the reference protein and processed in parallel.

#### Quantitative real-time PCR (qPCR)

RNA extraction from each cell group (group NC, NI, YTHDF2-kd, YTHDF2-kd+NI) was performed using TRIzol reagent (Sigma, 15596026). Subsequently, 1 μg of total RNA was reverse transcribed into cDNA using the ReverTra Ace qPCR RT kit (Vazyme), with genomic DNA removed. Quantitative PCR was conducted with SYBR Green qPCR master mix (Vazyme), using GAPDH as the internal control, on a real-time fluorescence PCR instrument. Each sample was analyzed three times.

#### Immunohistochemical analysis (IHC)

Tissue sections (4 μm thick) preserved in formalin and embedded in paraffin were deparaffinized using xylene and subsequently hydrated through a graded ethanol series. Endogenous peroxidase activity was blocked by incubating sections in 3% hydrogen peroxide for 10 min. Cells were incubated in 5% bovine serum albumin (BSA) for 30 min following PBS washing to reduce non-specific binding. Primary antibodies against YTHDF2 were applied to the sections and incubated overnight at 4°C. The biotin-labeled IgG secondary antibody was bound for 30 min at room temperature, followed by treatment with a streptavidin-horseradish peroxidase (HRP) complex.3,3′-Diaminobenzidine (DAB) served as the substrate for the color reaction. Sections were counterstained with hematoxylin, dehydrated, and sealed. Tissues were imaged using a confocal laser microscope, and images were taken for analysis.

#### Transwell migration assay

Cell suspensions were prepared and inoculated at a density of 2 × 10^∧^4 in Transwell chambers with polycarbonate membranes on 24-well plates. The lower chamber’s discarded medium was supplemented with varying drug concentrations, while the upper chamber received medium at double the concentration of the lower chamber. After 12 to 18 h, the liquid in the upper chamber was discarded. The chambers were then fixed with paraformaldehyde for 20 min, dried, and stained with crystal violet for another 20 min. The chambers were washed in clean water, the excess crystal violet was dried, and the chambers were photographed and counted.

#### Cell viability measurement

Cell proliferation was assessed using the Cell Counting Kit-8. Cells were inoculated in 96-well plates at a density of 1 × 10ˆ5 cells per well. When the cells reach a confluence of 50%–60%, discard the original culture medium and add the culture medium containing 16 μmol/L nicotinamide for treatment respectively. Following a 24-h incubation at 37°C with 5% CO2, the medium was replaced with one containing CCK-8 reagent for 90 min.Absorbance at 450 nm was recorded using a SpectraMax 190 microplate reader (Molecular Devices).

#### Colony formation assay

The trypsin-digested cell suspension was counted, resuspended, and inoculated at 5000 cells per well in six-well plates with 2 mL of fresh medium added. The trypsin-digested cell suspension was counted, resuspended, and seeded at 5000 cells per well in six-well plates with 2 mL of fresh medium added. When obvious aggregation occurs in the cells, add niclosamide (16 μmol/L). Replace the culture medium containing the drug every day and cultivate the petri dishes for 5–7 days until a clear trend appears in each Petri dish. The medium was discarded, washed with PBS, fixed using 1 mL 4% paraformaldehyde, and subsequently stained with 1 mL crystal violet solution, washed, and allowed to dry and photographed.

#### MTT assay

Post-digestion, cells were inoculated in 96-well plates at 5000 cells per well and incubated for 24 h at 37°C with 5% CO_2_. Once the cells reached 50%–60% confluence, they were treated with media containing 16 μmol/L niclosamide and incubated for 24 h. After a 4-h incubation with MTT solution in the well plate, the supernatant was removed, methylene solubilization solution was added, and absorbance was measured at 570 nm using a microplate reader.

#### Apoptosis assays

Annexin V-FITC Apoptosis Detection Kit was used to detect cell apoptosis.Beyotime) for apoptosis detection. Cells were digested with trypsin without EDTA in centrifuge tubes, followed by centrifugation and resuspension in Annexin V-FITC binding solution. The resuspension was transferred to the appropriate EP tube, with two single staining tubes and one negative control prepared, and staining was conducted at room temperature in the dark.The samples were detected sequentially with the corresponding channels.

#### Extracellular acidifcation rate (ECAR)

To evaluate the effects of niclosamide on sugar metabolism level, Seahorse XFe96 analyzer was performed to measure ECAR of BGC-823 cells. In brief, BGC-823 cells were seeded into Seahorse 96-well cell culture plate at cell density of 2×10^∧^4 and 2.1×10^∧^4 cells per well. Then, cells were pretreated with niclosamide (8 μM) for 24 h. Simultaneously, Seahorse probe plate was placed in a CO2-free, 37°C incubator for hydration and OCR assay medium was prepared (0.225 g D-glucose, 1 mL pyruvate sodium and 49 mL base medium, pH7.4). Next, glucose (10 mM), oligomycin (1 μM), or 2-DG (100 mM) were sequentially injected into each well. Overall ECAR of the cells was measured by seahorse XFe96 analyzer and plotted by Wave software. Each cell group includes at least 3 replicates.

#### Reactive oxygen species

Intracellular ROS levels were measured using a reactive oxygen species (ROS) assay kit (S0033S, Beyotime). BGC-823 cells were seeded into 6-well plates at a density of 5 × 10^∧^5 cells/well, with three replicates per group. When the cells density reached approximately 80%, treated them with niclosamide at concentrations of 0, 8, 16, and 32 μmol/L respectively. Cultivate under complete DMEM medium at 37 °C with 5% CO_2_ for 24 h. Cells were then harvested by trypsinization, transferred to centrifuge tubes, and subjected to probe loading and binding. The cells were stained at 37 °C for 30 min, washed three times with PBS, and subsequently analyzed using a flow cytometer (Agilent).

#### L- lactate measurement

Intracellular lactate levels were measured using a lactate assay kit (SNM184, Biorab, China) based on a colorimetric method. HMEC-1 endothelial cells were seeded into 6-well plates at a density of 2 × 10^∧^5 cells/well. When the cells density reached approximately 90%, they were treated with L-lactate at concentrations of 0, 4, 6, and 8 mmol/L in complete HMEC-1 medium (ZQ-1319, Zhongqiao Xinzhou, China) and incubated for 4 h. Cells were then harvested by trypsinization, centrifuged at 1200 rpm for 3 min, and resuspended in PBS as the homogenization medium. After ultrasonic disruption, the supernatant was collected and subjected to lactate measurement according to the manufacturer’s instructions.

### Quantification and statistical analysis

GraphPad Prism (v 8.0.2, GraphPad, USA) was used to plot the data. YTHDF2 expression was compared between normal and tumor samples using the Wilcoxon signed-rank test, and among multiple groups using one-way ANOVA. Statistical significance was denoted as: ∗*p* < 0.05, ∗∗*p* < 0.01, ∗∗∗*p* < 0.001, ∗∗∗∗*p* < 0.0001, with ‘n.s.’ indicating no significance. All experiments in this study were conducted with biological replicates.
