# Supplementary material for: Niclosamide suppresses gastric cancer progression through YTHDF2 inhibition-affected lactate metabolic reprogramming
Source: iScience. 2025 Oct 27;28(11):113868. doi: 10.1016/j.isci.2025.113868 (PMC12661202; doi:10.1016/j.isci.2025.113868)
Supplement: Document S1. Figures S1–S6 [file mmc1.pdf]

iScience, Volume *28*

Supplemental information

Niclosamide suppresses gastric cancer progression through YTHDF2 inhibition-affected lactate metabolic reprogramming

Mosheng Tang, Yiyin Hu, Xiaohui Zhu, Jichen Dai, Rui Li, Xinran Feng, Kangrui Chi, Xinyi Li, Qiaofei Zhang, Ke Chen, Ruoying Cao, Hanqing Zhu, Haoran Huang, Haipeng Wu, Lejia Sun, Tongke Chen, Jun Wu, Huafu Wang, and Zhihong Gui

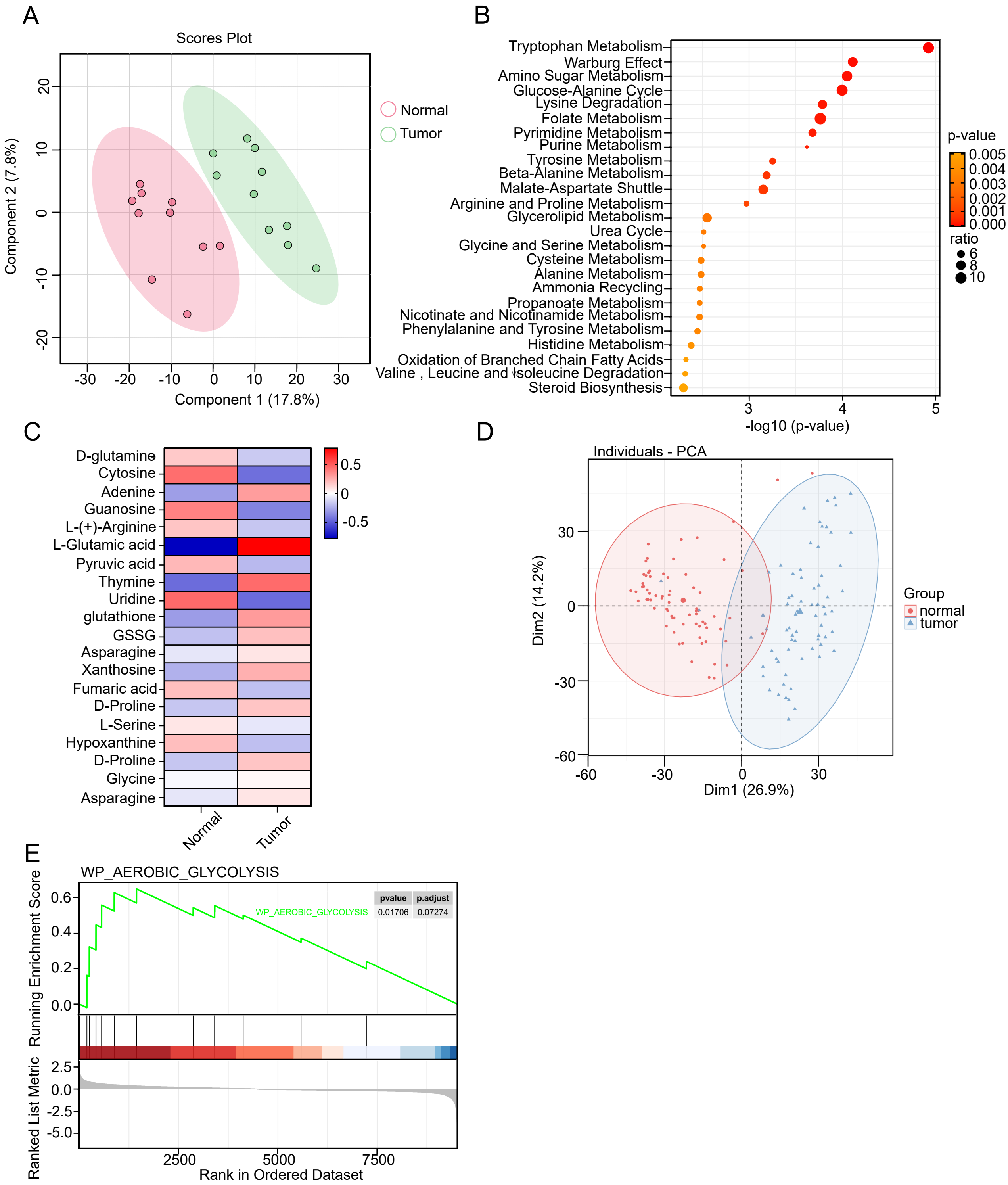

A
Scores Plot
Component 1 (17.8%)
Component 2 (7.8%)
Normal
Tumor
B
Tryptophan Metabolism
Warburg Effect
Amino Sugar Metabolism
Glucose-Alanine Cycle
Lysine Degradation
Folate Metabolism
Pyrimidine Metabolism
Purine Metabolism
Tyrosine Metabolism
Beta-Alanine Metabolism
Malate-Aspartate Shuttle
Arginine and Proline Metabolism
Glycerolipid Metabolism
Urea Cycle
Glycine and Serine Metabolism
Cysteine Metabolism
Alanine Metabolism
Ammonia Recycling
Propanoate Metabolism
Nicotinate and Nicotinamide Metabolism
Phenylalanine and Tyrosine Metabolism
Histidine Metabolism
Oxidation of Branched Chain Fatty Acids
Valine , Leucine and Isoleucine Degradation
Steroid Biosynthesis
-log10 (p-value)
p-value
ratio
C
D-glutamine
Cytosine
Adenine
Guanosine
L-(+)-Arginine
L-Glutamic acid
Pyruvic acid
Thymine
Uridine
glutathione
GSSG
Asparagine
Xanthosine
Fumaric acid
D-Proline
L-Serine
Hypoxanthine
D-Proline
Glycine
Asparagine
Normal
Tumor
D
Individuals - PCA
Dim1 (26.9%)
Dim2 (14.2%)
Group
normal
tumor
E
WP_AEROBIC_GLYCOLYSIS
Running Enrichment Score
Ranked List Metric
Rank in Ordered Dataset
pvalue p.adjust
WP_AEROBIC_GLYCOLYSIS 0.01706 0.07274

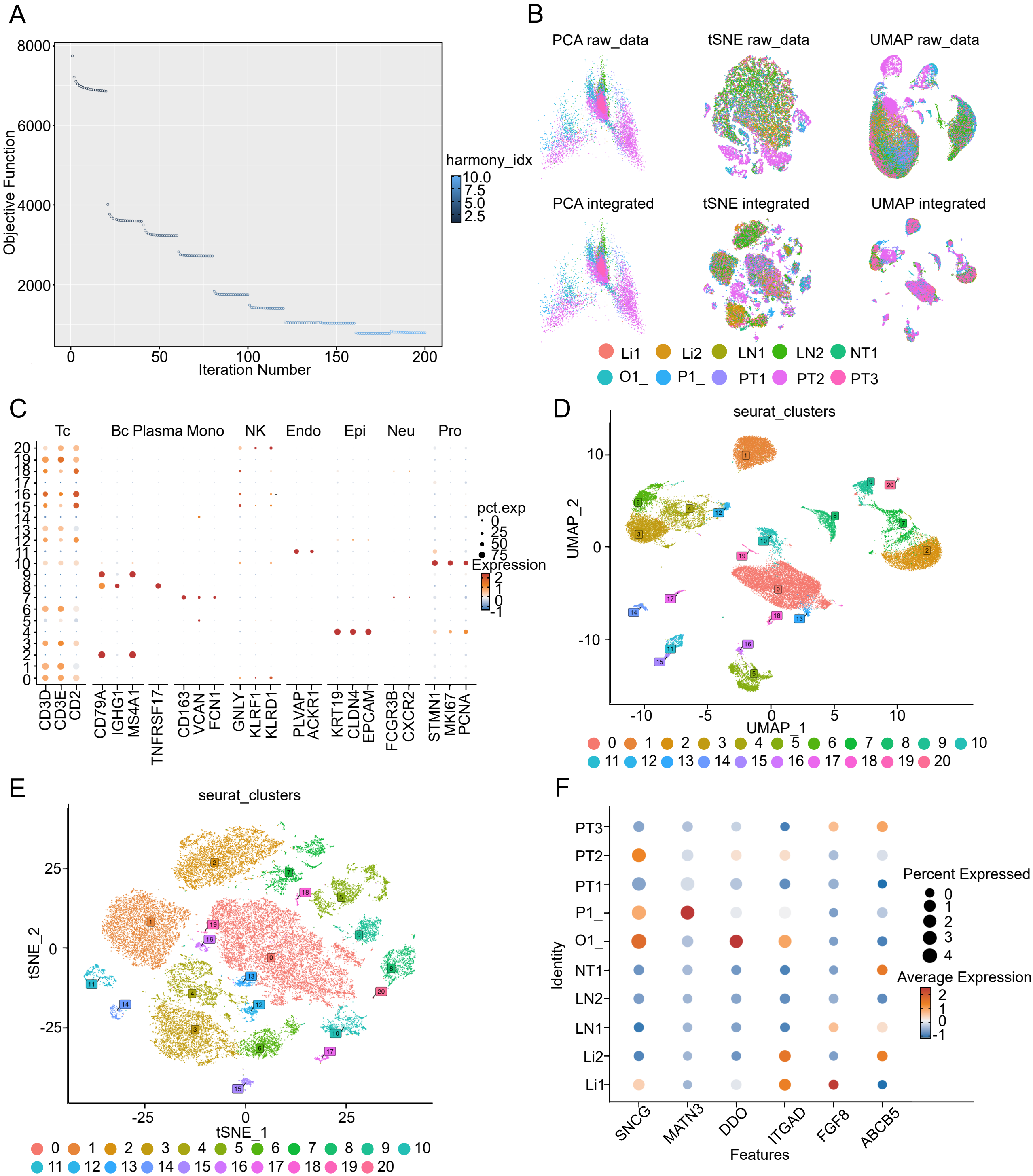
A
Objective Function
Iteration Number
harmony_idx
10.0
7.5
5.0
2.5
8000
6000
4000
2000
0
50
100
150
200
B
PCA raw_data
tSNE raw_data
UMAP raw_data
PCA integrated
tSNE integrated
UMAP integrated
Li1
Li2
LN1
LN2
NT1
O1_
P1_
PT1
PT2
PT3
C
Tc
Bc Plasma Mono
NK
Endo
Epi
Neu
Pro
pct.exp
0
25
50
75
Expression
2
1
0
-1
CD3D
CD3E
CD2
CD79A
IGHG1
MS4A1
TNFRSF17
CD163
VCAN
FCN1
GNLY
KLRF1
KLRD1
PLVAP
ACKR1
KRT19
CLDN4
EPCAM
FCGR3B
CXCR2
STMN1
MKI67
PCNA
D
seurat_clusters
UMAP_1
UMAP_2
10
0
-10
-5
5
E
seurat_clusters
tSNE_1
tSNE_2
25
0
-25
F
PT3
PT2
PT1
P1_
O1_
NT1
LN2
LN1
Li2
Li1
Identity
SNCG
MATN3
DDO
ITGAD
FGF8
ABCB5
Features
Percent Expressed
0
1
2
3
4
Average Expression
2
1
0
-1

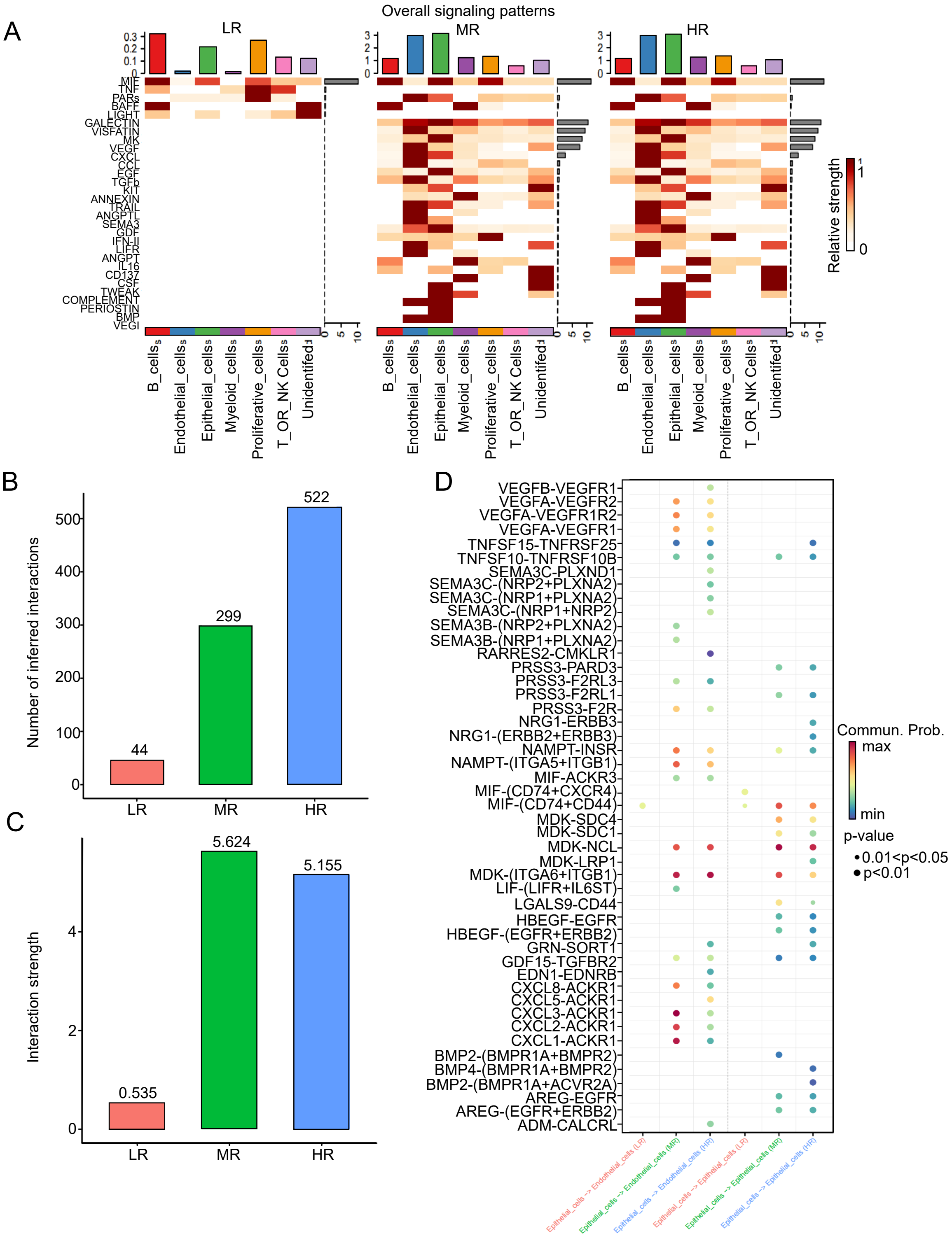

Overall signaling patterns
A
LR
MR
HR
MIF
TNF
PARs
BAFF
LIGHT
GALECTIN
VISFATIN
MK
VEGF
CXCL
CCL
EGF
TGFb
KIT
ANNEXIN
TRAIL
ANGPTL
SEMA3
GDF
IFN-II
LIFR
ANGPT
IL16
CD137
CSF
TWEAK
COMPLEMENT
PERIOSTIN
BMP
VEGI
B_cells
Endothelial_cells
Epithelial_cells
Myeloid_cells
Proliferative_cells
T_OR_NK Cells
Unidentifed
Relative strength
B
Number of inferred interactions
44
299
522
LR
MR
HR
C
Interaction strength
0.535
5.624
5.155
D
VEGFB-VEGFR1
VEGFA-VEGFR2
VEGFA-VEGFR1R2
VEGFA-VEGFR1
TNFSF15-TNFRSF25
TNFSF10-TNFRSF10B
SEMA3C-PLXND1
SEMA3C-(NRP2+PLXNA2)
SEMA3C-(NRP1+PLXNA2)
SEMA3C-(NRP1+NRP2)
SEMA3B-(NRP2+PLXNA2)
SEMA3B-(NRP1+PLXNA2)
RARRES2-CMKLR1
PRSS3-PARD3
PRSS3-F2RL3
PRSS3-F2RL1
PRSS3-F2R
NRG1-ERBB3
NRG1-(ERBB2+ERBB3)
NAMPT-INSR
NAMPT-(ITGA5+ITGB1)
MIF-ACKR3
MIF-(CD74+CXCR4)
MIF-(CD74+CD44)
MDK-SDC4
MDK-SDC1
MDK-NCL
MDK-LRP1
MDK-(ITGA6+ITGB1)
LIF-(LIFR+IL6ST)
LGALS9-CD44
HBEGF-EGFR
HBEGF-(EGFR+ERBB2)
GRN-SORT1
GDF15-TGFBR2
EDN1-EDNRB
CXCL8-ACKR1
CXCL5-ACKR1
CXCL3-ACKR1
CXCL2-ACKR1
CXCL1-ACKR1
BMP2-(BMPR1A+BMPR2)
BMP4-(BMPR1A+BMPR2)
BMP2-(BMPR1A+ACVR2A)
AREG-EGFR
AREG-(EGFR+ERBB2)
ADM-CALCRL
Epithelial_cells -> Endothelial_cells (LR)
Epithelial_cells -> Endothelial_cells (MR)
Epithelial_cells -> Endothelial_cells (HR)
Epithelial_cells -> Epithelial_cells (LR)
Epithelial_cells -> Epithelial_cells (MR)
Epithelial_cells -> Epithelial_cells (HR)
Commun. Prob.
max
min
p-value
0.01<p<0.05
p<0.01

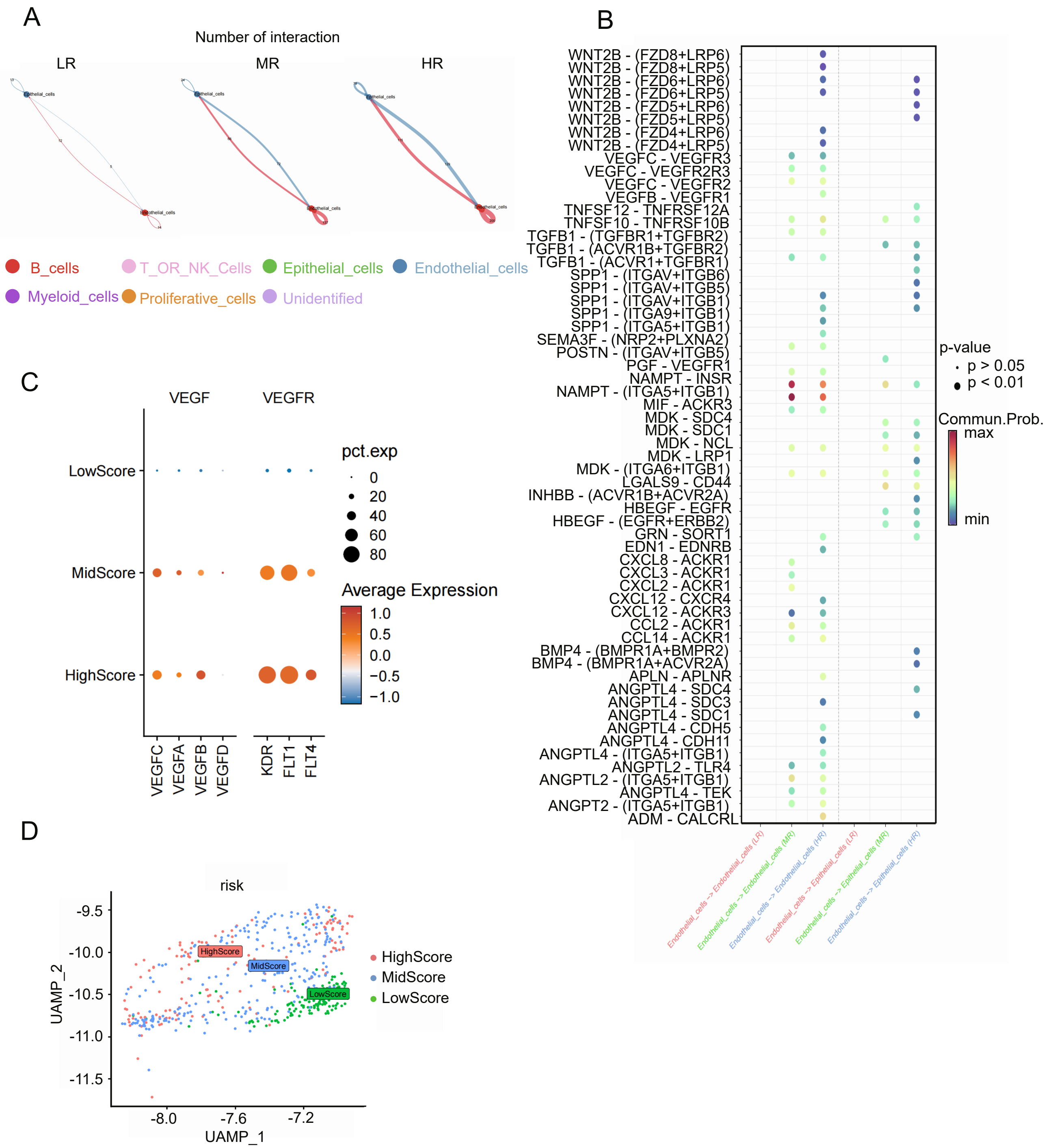

A
Number of interaction
LR
MR
HR
B_cells
T_OR_NK_Cells
Epithelial_cells
Endothelial_cells
Myeloid_cells
Proliferative_cells
Unidentified
B
WNT2B - (FZD8+LRP6)
WNT2B - (FZD8+LRP5)
WNT2B - (FZD6+LRP6)
WNT2B - (FZD6+LRP5)
WNT2B - (FZD5+LRP6)
WNT2B - (FZD5+LRP5)
WNT2B - (FZD4+LRP6)
WNT2B - (FZD4+LRP5)
VEGFC - VEGFR3
VEGFC - VEGFR2R3
VEGFC - VEGFR2
VEGFB - VEGFR1
TNFSF12 - TNFRSF12A
TNFSF10 - TNFRSF10B
TGFB1 - (TGFBR1+TGFBR2)
TGFB1 - (ACVR1B+TGFBR2)
TGFB1 - (ACVR1+TGFBR1)
SPP1 - (ITGAV+ITGB6)
SPP1 - (ITGAV+ITGB5)
SPP1 - (ITGAV+ITGB1)
SPP1 - (ITGA9+ITGB1)
SPP1 - (ITGA5+ITGB1)
SEMA3F - (NRP2+PLXNA2)
POSTN - (ITGAV+ITGB5)
PGF - VEGFR1
NAMPT - INSR
NAMPT - (ITGA5+ITGB1)
MIF - ACKR3
MDK - SDC4
MDK - SDC1
MDK - NCL
MDK - LRP1
MDK - (ITGA6+ITGB1)
LGALS9 - CD44
INHBB - (ACVR1B+ACVR2A)
HBEGF - EGFR
HBEGF - (EGFR+ERBB2)
GRN - SORT1
EDN1 - EDNRB
CXCL8 - ACKR1
CXCL3 - ACKR1
CXCL2 - ACKR1
CXCL12 - CXCR4
CXCL12 - ACKR3
CCL2 - ACKR1
CCL14 - ACKR1
BMP4 - (BMPR1A+BMPR2)
BMP4 - (BMPR1A+ACVR2A)
APLN - APLNR
ANGPTL4 - SDC4
ANGPTL4 - SDC3
ANGPTL4 - SDC1
ANGPTL4 - CDH5
ANGPTL4 - CDH11
ANGPTL4 - (ITGA5+ITGB1)
ANGPTL2 - TLR4
ANGPTL2 - (ITGA5+ITGB1)
ANGPTL4 - TEK
ANGPT2 - (ITGA5+ITGB1)
ADM - CALCRL
Endothelial_cells -> Endothelial_cells (LR)
Endothelial_cells -> Endothelial_cells (MR)
Endothelial_cells -> Endothelial_cells (HR)
Endothelial_cells -> Epithelial_cells (LR)
Endothelial_cells -> Epithelial_cells (MR)
Endothelial_cells -> Epithelial_cells (HR)
p-value
p > 0.05
p < 0.01
Commun.Prob.
max
min
C
VEGF
VEGFR
LowScore
MidScore
HighScore
VEGFC
VEGFA
VEGFB
VEGFD
KDR
FLT1
FLT4
pct.exp
0
20
40
60
80
Average Expression
1.0
0.5
0.0
−0.5
−1.0
D
risk
HighScore
MidScore
LowScore
UAMP_2
UAMP_1
-9.5
-10.0
-10.5
-11.0
-11.5
-8.0
-7.6
-7.2

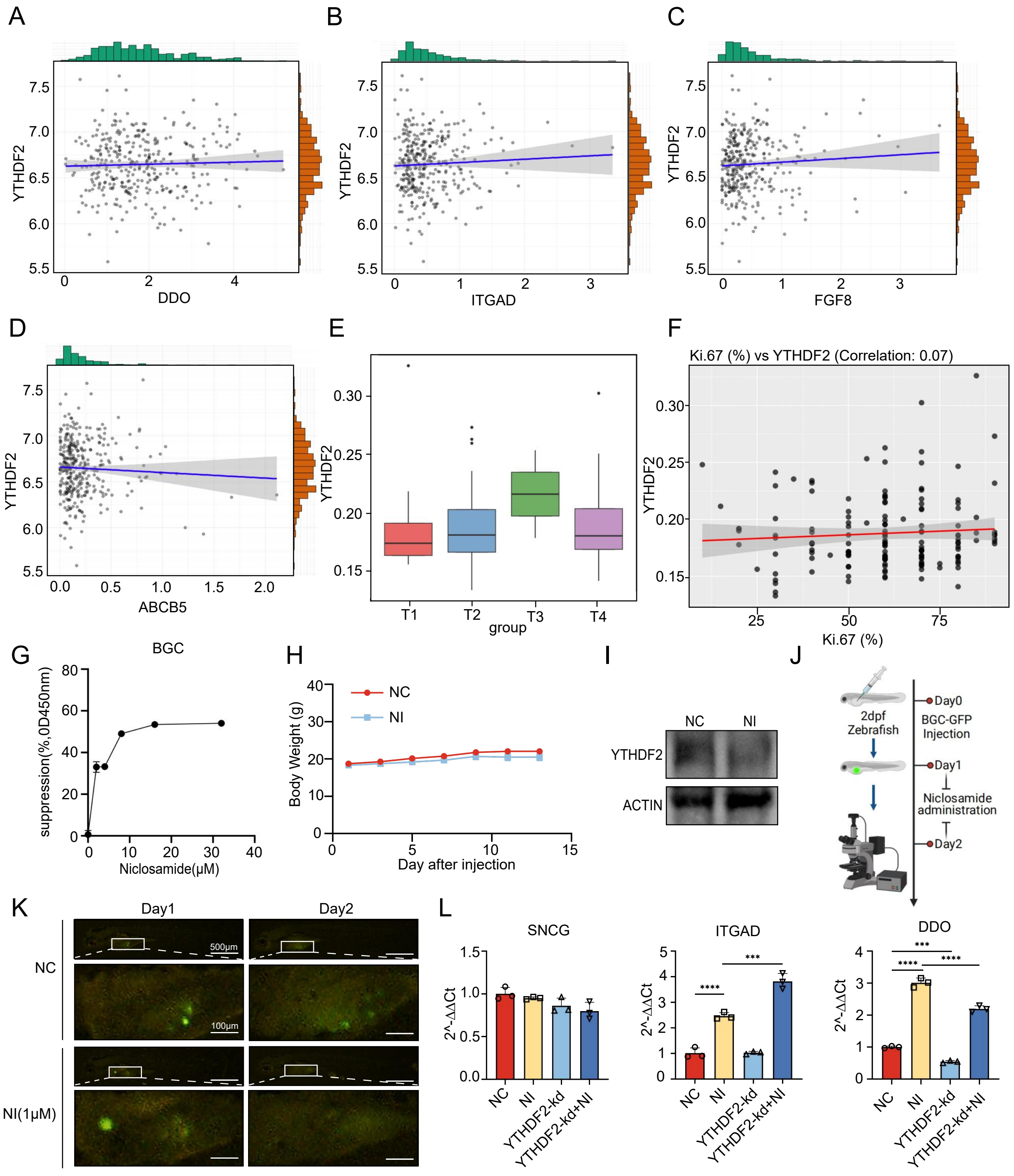

A
YTHDF2
DDO
B
YTHDF2
ITGAD
C
YTHDF2
FGF8
D
YTHDF2
ABCB5
E
YTHDF2
T1
T2
T3
T4
group
F
Ki.67 (%) vs YTHDF2 (Correlation: 0.07)
YTHDF2
Ki.67 (%)
G
BGC
suppression(%,0D450nm)
Niclosamide(μM)
H
NC
NI
Body Weight (g)
Day after injection
I
NC
NI
YTHDF2
ACTIN
J
2dpf
Zebrafish
Day0
BGC-GFP
Injection
Day1
Niclosamide
administration
Day2
K
Day1
Day2
NC
NI(1μM)
500μm
100μm
L
SNCG
ITGAD
DDO
2^-ΔΔCt
NC
NI
YTHDF2-kd
YTHDF2-kd+NI

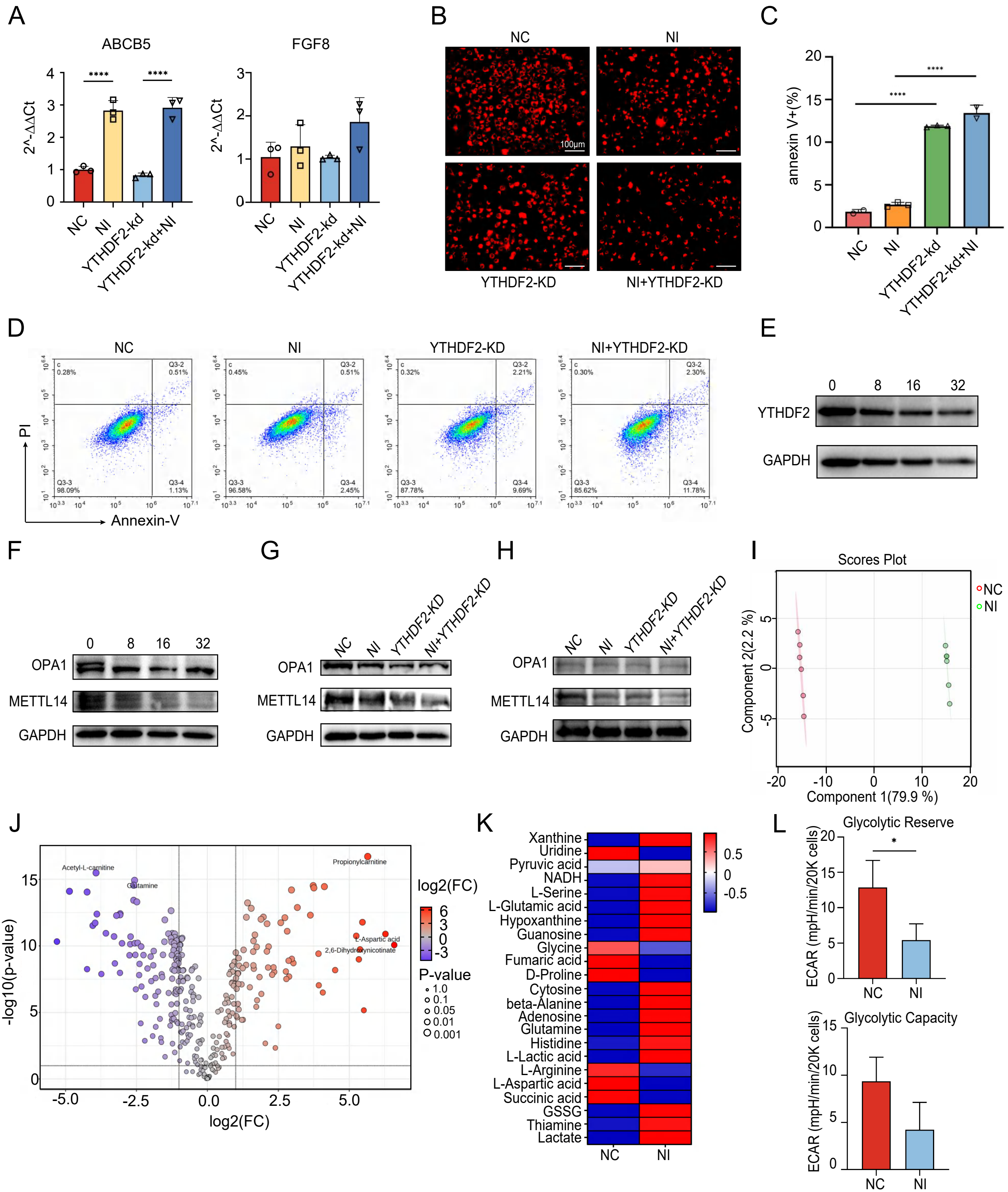

A
ABCB5
FGF8
2^-ΔΔCt
****
NC
NI
YTHDF2-kd
YTHDF2-kd+NI
B
NC
NI
100μm
YTHDF2-KD
NI+YTHDF2-KD
C
annexin V+(%)
D
NC
NI
YTHDF2-KD
NI+YTHDF2-KD
PI
Annexin-V
E
0
8
16
32
YTHDF2
GAPDH
F
OPA1
METTL14
GAPDH
G
H
I
Scores Plot
NC
NI
Component 2(2.2 %)
Component 1(79.9 %)
J
Acetyl-L-carnitine
Glutamine
Propionylcarnitine
L-Aspartic acid
2,6-Dihydroxynicotinate
log2(FC)
P-value
-log10(p-value)
K
Xanthine
Uridine
Pyruvic acid
NADH
L-Serine
L-Glutamic acid
Hypoxanthine
Guanosine
Glycine
Fumaric acid
D-Proline
Cytosine
beta-Alanine
Adenosine
Glutamine
Histidine
L-Lactic acid
L-Arginine
L-Aspartic acid
Succinic acid
GSSG
Thiamine
Lactate
L
Glycolytic Reserve
Glycolytic Capacity
ECAR (mpH/min/20K cells)

Figure S1 [Gastric cancer patients have global changes in metabolism.] Related to Figure 1

A. Partial least-squares discriminant analysis (PLSDA) of normal and tumor tissues.

B. The bubble plot shows the enrichment of metabolic pathways in tumor tissue.

C. Differences in levels of metabolites in normal and tumor tissues.

D. PCA analysis of normal and tumor tissues

E. Gene set enrichment analysis (GSEA) of the WP_AEROBIC_GLYCOLYSIS pathway.

Figure S2 [Integration and clustering analysis of single-cell RNA-seq data.] Related to Figure 4

A. Convergence curve of the iterative optimization process, showing the convergence of the Harmony algorithm over 200 iterations, with the color scale representing the Harmony index.

B. Dimensionality reduction visualizations of raw and integrated data using PCA, t-SNE, and UMAP.

C. Annotated dot plot of cell types and related expression metrics, displaying the expression levels of marker genes in different cell types.

D. UMAP plot of cells colored by Seurat clusters, showing the distribution of distinct cell populations.

E. t-SNE plot of cells colored by Seurat clusters, providing an alternative view of the cluster organization.

F. Feature expression heatmap showing the percentage of cells expressing selected genes (SNCA, MATN, DDO, TGAD, FGF8, ABCB5) across different groups.

Figure S3 [Integration and clustering analysis of single-cell RNA-seq data.] Related to figure 5

A. Heatmaps depicting the relative signaling patterns for Low-risk (LR), Medium-risk (MR), and High-risk (HR) groups.

B. Bar plot shows the total number of interactions in each risk group, with a marked increase in interaction number from LR to HR.

C. Bar plot shows the interaction strength for each group, showing higher interaction strength in MR and HR compared to LR.

D. Network of significant protein-protein interactions (PPIs) identified in each group.

Figure S4 [Overview of signaling patterns and interaction strengths in different groups.] Related to figure 5

A. Directed graphs shows the number of interactions between specific cell types in LR, MR, and HR groups.

B. Dot plot shows the communication probability between various cell type interactions, with statistical significance indicated by color.

C. The expression levels of VEGF and VEGFR in endothelial cells.

D. The UMAP of endothelial cells representing 3 unique states color-coded by their risk score.

Figure S5 [Correlation between YTHDF2 and metabolic genes.] Related to figure 7

A. Correlation analysis of YTHDF2 and DDO.

B. Correlation analysis of YTHDF2 and ITGAD.

C. Correlation analysis of YTHDF2 and FGF8.

D. Correlation analysis of YTHDF2 and ABCB5.

E. The box plot showed the expression of YTHDF2 in different tumor stages.

F. Correlation analysis of YTHDF2 and DDO.

G. The CCK8 assay in BGC-823 cells.

H. The body weights of BALB/c-nu mice in control group and Niclosamide treatment group (n=3).

I. Western Blot detection of YTHDF2 in tumor tissues of BALB/c-nu mice.

J. Schematic diagram of the tumor model in zebrafish being treated with niclosamide (1μM), created by Biorender.

K. Fluorescence changes of tumor cells in zebrafish treated with niclosamide for two days. Scale bar, 500μm and 100μm.

L. Real-time qPCR analysis for SNCG, ITGAD, DDO expression (***P<0.001, ****P<0.0001). Data are represented as mean ± SD.

Figure S6 [Effects of Niclosamide and YTHDF2 knockdown on cell and metabolism.] Related to figure 7、8

A. Real-time qPCR analysis for FGF8, ABCB5 expression (***P<0.001, ****P<0.0001). Data are represented as mean ± SD.

B. The effect of Niclosamide and YTHDF2 knockdown on mitochondrial fluorescence intensity. Scale bar, 100μm.

C. The quantitative analysis of the impact of Niclosamide and YTHDF2 knockdown on apoptosis in gastric cancer cells was conducted (****$P<0.0001$). Data are represented as mean ± SD.

D. Apoptotic effect of Niclosamide and YTHDF2 knockdown in BGC-823 cells.

E-H. Western blot detection of YTHDF2, OPA1 and METT14.

J . Partial least-squares discriminant analysis (PLSDA) of NC and Ni.

J . Volcano plot depicting significant abundant metabolites between NC and Ni. Metabolites are highlighted if they have a p-value < 0.05 and |log2 FC| > 1.

K . Differences in levels of metabolites in NC and Ni.

L . The Glycolytic Reserve (*$P<0.05$) and Glycolytic Capacity of the control group and the Niclosamide treatment group. Data are represented as mean ± SD.
